# Piperazinyl fragment improves anticancer activity of Triapine

**DOI:** 10.1371/journal.pone.0188767

**Published:** 2018-04-13

**Authors:** Marta Rejmund, Anna Mrozek-Wilczkiewicz, Katarzyna Malarz, Monika Pyrkosz-Bulska, Kamila Gajcy, Mieczyslaw Sajewicz, Robert Musiol, Jaroslaw Polanski

**Affiliations:** 1 Institute of Chemistry, University of Silesia, Katowice, Poland; 2 A. Chełkowski Institute of Physics, University of Silesia, Katowice, Poland; 3 Silesian Center for Education and Interdisciplinary Research, University of Silesia, Chorzów, Poland; Virginia Commonwealth University, UNITED STATES

## Abstract

A new class of TSCs containing piperazine (piperazinylogs) of Triapine, was designed to fulfill the di-substitution pattern at the TSCs N4 position, which is a crucial prerequisite for the high activity of the previously obtained TSC compounds–DpC and Dp44mT. We tested the important physicochemical characteristics of the novel compounds L^1^-L^12^. The studied ligands are neutral at physiological pH, which allows them to permeate cell membranes and bind cellular Fe pools more readily than less lipid-soluble ligands, e.g. DFO. The selectivity and anti-cancer activity of the novel TSCs were examined in a variety of cancer cell types. In general, the novel compounds demonstrated the greatest promise as anti-cancer agents with both a potent and selective anti-proliferative activity. We investigated the mechanism of action more deeply, and revealed that studied compounds inhibit the cell cycle (G1/S phase). Additionally we detected apoptosis, which is dependent on cell line’s specific genetic profile. Accordingly, structure-activity relationship studies suggest that the combination of the piperazine ring with Triapine allows potent and selective anticancer chelators that warrant further *in vivo* examination to be identified. Significantly, this study proved the importance of the di-substitution pattern of the amine N4 function.

## Introduction

Thiosemicarbazones (TSCs) have a broad range of biological activity including antitumor, antimalarial and antimicrobial activity [1], and therefore, for many years, studies of α-(N)-heterocyclic TSCs have been attracting considerable interest. In particular, the antitumor properties of 2-formylpyridine thiosemicarbazone were reported over 50 years ago [2]. With regard to potential pharmaceutical applications, Triapine (3-aminopyridine-2-carboxaldehyde thiosemicarbazone; 3-AP) is the most prominent representative of this class, as it has already been investigated in more than 30 clinical phase I/II trials [3–10]. Moreover, di-2-pyridylketone-4-cyclohexyl-4methyl-3-thiosemicarbazone (DpC) is currently entering clinical phase I studies as a potential anticancer agent. Although clinical studies have concluded that Triapine revealed activity against hematological cancer types (e.g. advanced leukemia [8,11]), it also showed disappointing results against a variety of solid tumor types such as advanced adenocarcinoma of the pancreas [12], non-small-cell lung cancer [3] and renal cell carcinoma [13]. Furthermore, some side effects include the formation of methemoglobin and hypoxia [3,8,9], which were observed after administration. Therefore, the development of new Triapine analogs with a potent anticancer activity would be significant.

The broad range of the biological activity of TSCs corresponds to their versatile binding modes with the transition and main group metal ions [14]. Moreover, it has been observed that, generally, the biological activity of the complexes of TSCs is often higher than that of corresponding metal-free ligands. To gain further insight into the coordination chemistry of TSCs, thermodynamic data such as the stability constants of metal complexes, which help in optimizing the chemical or biological properties that are essential for potential medicinal applications, are needed. In particular, copper complexes have a considerably higher anticancer activity than the uncomplexed ligands that also have lower IC_50_ values against cancer cells than other described topoisomerase-II inhibitors [15]. In contrast, Triapine complexation to iron resulted in the reduced cytotoxicity compared to the metal free ligand.

Although Triapine, similar to the recently developed Dp44mT (di-2-pyridylketone-4,4-dimethyl-3-thiosemicarbazone), has been evaluated as a potential anticancer agent, the molecular mechanisms of its action have not been fully elucidated. Several paradigms have been proposed to explain the activity of these compounds [16,17] including blocking cellular iron uptake from transferrin [18]; mobilizing iron from cells; inhibiting ribonucleotide reductase, the iron-containing enzyme that is involved in the rate-limiting step of DNA synthesis [19] or forming reactive oxygen species (ROS) [20]. The effect of metal chelation suggested among potential determinants of the mechanism origins are among the most important determiners deciding that the mechanism(s) of action of these compounds are incompletely understood.

**Fig 1:**
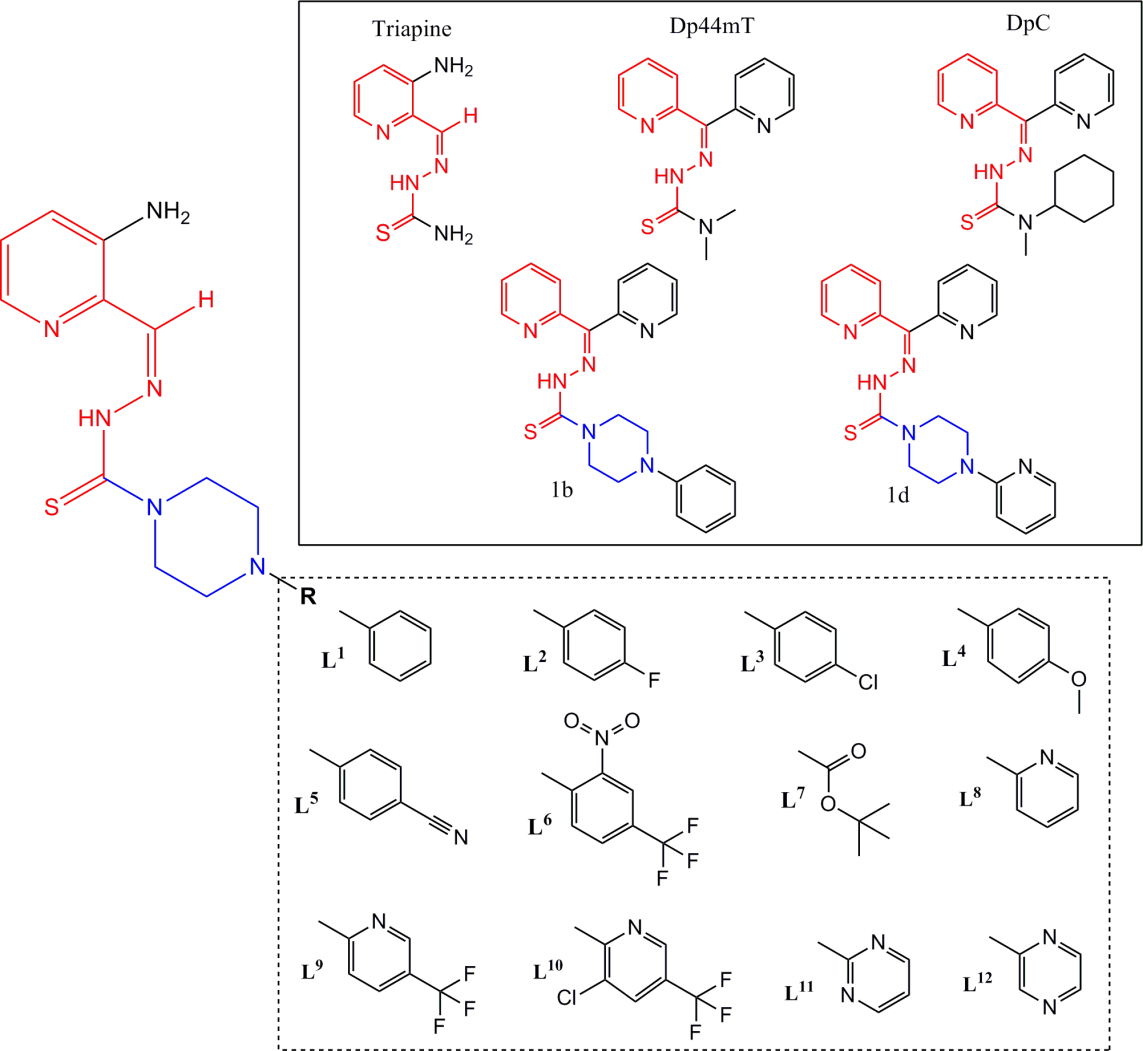
Design strategy for novel TSCs (L^1^-L^12^). All designed ligands are based on the Triapine skeleton, which is present in the active analogs Dp44mT, DpC and 1b, 1d that have been described as highly active analogs [21].

In this research, we designed and obtained new Triapine analogs for the first time through incorporating the piperazine ring as a promising new pharmacophore group to replace the N terminal amino group (Fig 1). While the substitution pattern at the N4 atom of TSCs appears to be critical for the activity of Dp44mT, there were no similar studies for Triapine. Therefore, we tested di-substitution at the N4 atom by constructing an N4-based piperazine, which is a fragment that is present in several active TSCs [21–23]. First, we hoped that this could modulate the antiproliferative activity of the new analogs because the piperazine heterocycle is found in a wide variety of biologically active compounds, some of which are currently being used in clinical therapy [24–27]. In particular, new derivatives could have a significant impact on pharmacokinetics and pharmacodynamics, while the replacement of the unsubstituted NH_2_ function with the piperazine fragment should increase the lipophilicity of the new analogs. Second, the modification of the substitution pattern of piperazine is a standard drug design scheme that has often resulted in an increased medicinal potential of the analogs [28].

Accordingly, we describe the synthesis of a series of new N4- piperazinylogs of Triapine that were tested for their anticancer activity vs. a broad spectrum of cancer cell lines. As the metal complexation of the piperazine ring has been reported as an important issue in the biological activity for these compounds [29,30], we tested L^1^-L^12^ as potential chelators for transition metals. Solution equilibria of the all the ligands and their copper(II) and iron(III) complexes were studied using UV-Vis titration.

## Results and discussion

### Chemistry

#### Design and synthesis

Identifying the functional fragments for drug design is a complex problem that involves different approaches including those that have an experimental and theoretical basis. The latter consist of a variety of methods, among which are those to identify advantageous sub-structures, scaffolds and/or linkers on the basis of previously reported compounds. Alternatively, the fragmentation of organic molecules into smaller moieties is an important method in retrosynthetic analysis and has inspired various pseudo-retrosynthetic approaches [31]. This has identified fragments that may be useful for drug design. For example, the di-2-pyridyl [32–35], quinolinyl [36], piperazinyl [37,38], morpholinyl [39] and quinoxalinyl [40] motifs, which are common fragments in other anti-cancer agents, have been incorporated into the design of the novel TSCs reported herein (Fig 1).

We have previously examined a variety of TSCs that demonstrate *in vitro* anti-proliferative activity [21,41,42]. Earlier studies indicated that di-substitution at the terminal (N4) nitrogen is crucial for effective anti-cancer activity [21,32,34,42]. Therefore, in the present study, we transformed the amine function in the southern part of the Triapine molecule into the form of a piperazine ring. This formed a di-substitution at the N4 position that was discovered to be of crucial importance for the activity of DpC and Dp44mT, which has never been tested for Triapine. In particular, there is a fragment that is present in several active TSCs in this piperazine ring [23,43].

**Fig 2:**
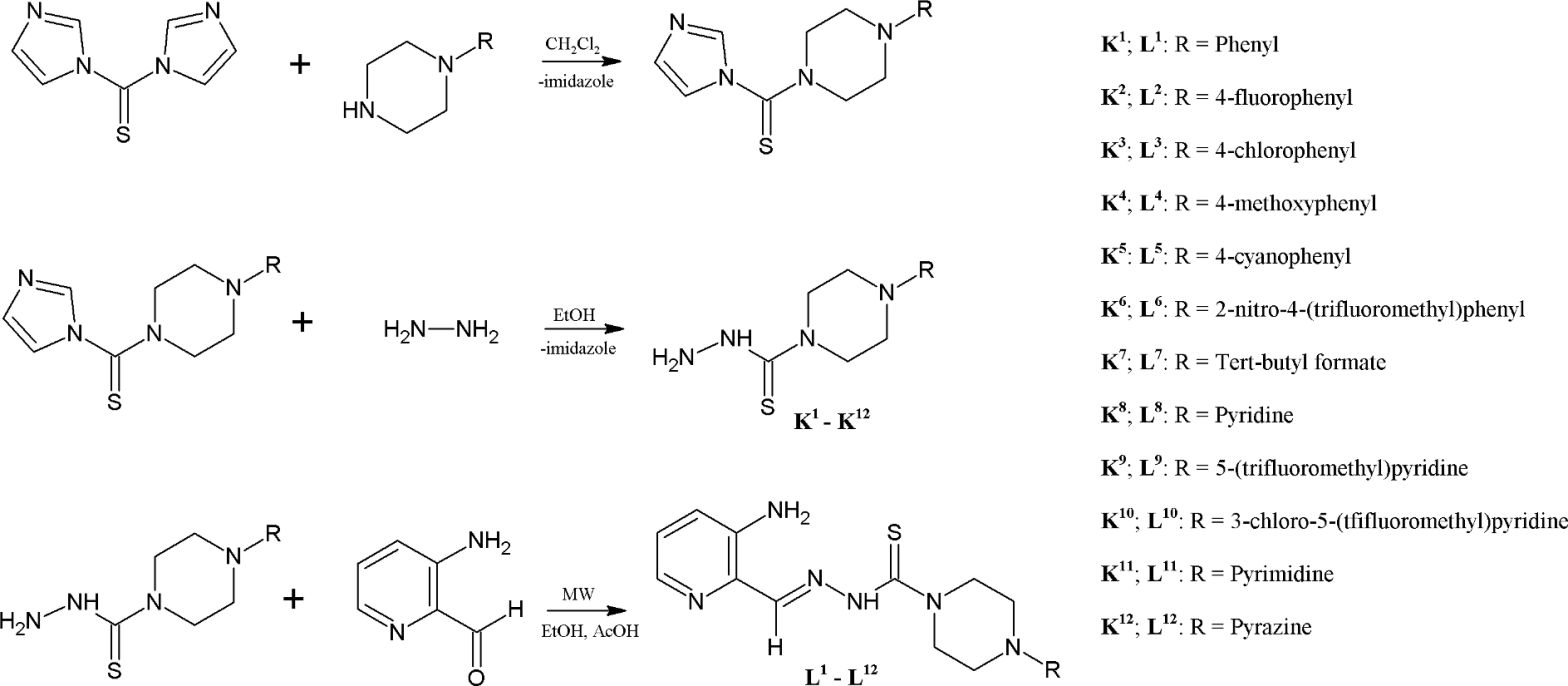
Synthesis of the thiosemicarbazides K^1^-K^12^ and thiosemicarbazones L^1^-L^12^.

The synthetic protocol that was used to produce the target molecules is outlined in Fig 2. The precursors that are required to obtain the desired Triapine derivatives, the thiosemicarbazides K^1^-K^12^, were synthesized from commercially available reagents in a two-step process that mostly produced high yields (69–98%). The treatment of (1,1’-thiocarbonyl)bis-1H-imidazole with the appropriate derivative of piperazine, followed by the reaction with hydrazine hydrate, produced the N-substituted piperazine-based thiosemicarbazides in a high yield. The final TSC series, L^1^-L^12^ (Fig 2), were synthesized in a moderate to high yield (20–83%) using the Schiff-based condensation of the 3-aminopyridine-2-carboxaldehyde with the prepared thiosemicarbazides K^1^-K^12^ in a microwave reactor, which produced novel Triapine-based ligands after crystallization with methanol. All the synthesized compounds were confirmed using ^1^H, ^13^C NMR and MS spectroscopic techniques.

### Chelating properties

#### Protonation constants of the Triapine-derivative ligands

**Fig 3:**
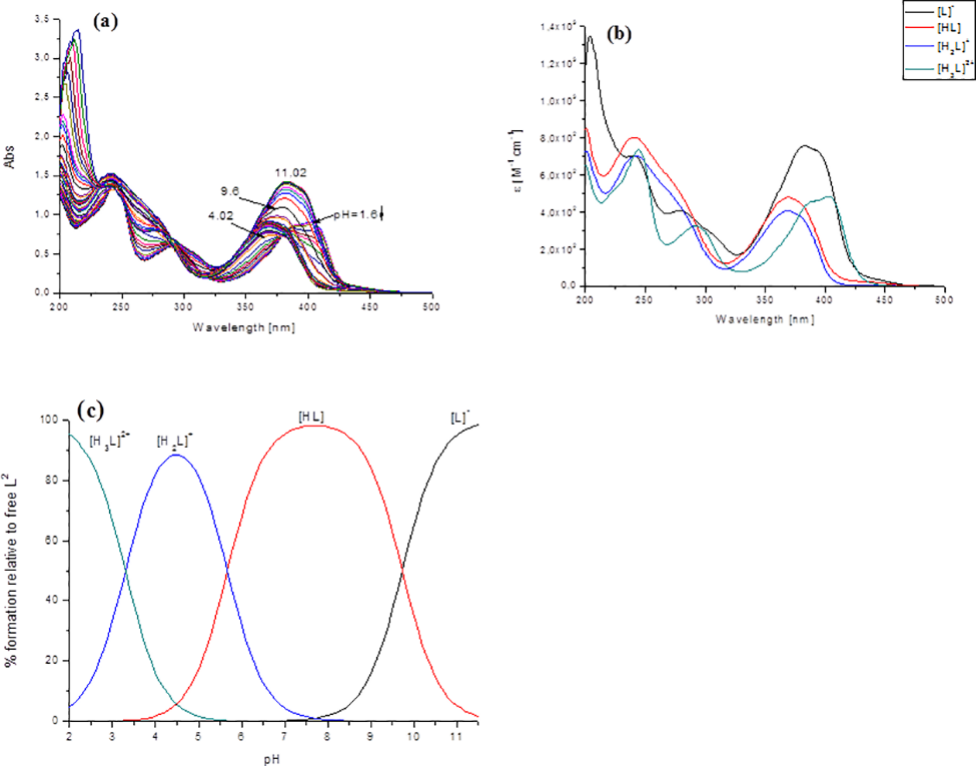
(a) Absorption spectrophotometric titration vs. pH of the free L^2^ ligand; (b) electronic spectra of the protonated species of L^2^; (c) concentration distribution curves for the L^2^ species. (I = 0.1 M (KCl) in 80% (w/w) MeOH/H_2_O; T = 25.0°C; [L^2^] = 5x10^-5^M; pH 1.6–11.02).

Spectrophotometric titrations were performed in order to probe the acid-based equilibria that were associated with each ligand and to determine the pH range over which the chelator was in its charge neutral form. This property is important in understanding the passage of a molecule through the cell membranes, as charged chelators have poor access [33,44]. These studies were performed in an 80% (w/w) MeOH/H_2_O solvent mixture due to the low solubility of these compounds in pure water. The fully protonated forms of the ligands have four L^1^-L^7^ or five L^8^-L^12^ dissociable protons, respectively. All the studied ligands possess one dissociable proton at the hydrazanic group of the thiosemicarbazone moiety, one at pyridine ring and two at the piperazine moiety. An additional proton in L^8^-L^12^ derives from the additional pyridine, pyrimidine or pyrazine ring, respectively; however, not all of them could be determined under these experimental conditions. The protonation constants that were obtained are given in Table 1 and the species distribution diagram of the protonated species of the selected ligand is presented in Fig 3.

**Table 1:** Protonation constants (log*β* ^H^) of the L^1^-L^12^ ligands in the MeOH/H_2_O mixed solution^a^.

	$\log\beta {}_{1}^{H}$	$\log\beta {}_{2}^{H}$	$\log\beta {}_{3}^{H}$	$\log\beta {}_{4}^{H}$	log*K*_*1*_	log*K*_*2*_	log*K*_*3*_	log*K*_*4*_
**L**^**1**^	9.67(1)	16.17(1)	19.49(1)		9.67	6.50	3.32	
**L**^**2**^	9.73 (1)	15.40 (2)	18.70(2)		9.73	5.77	3.20	
**L**^**3**^	9.25(1)	15. 83(2)	19.05(2)		9.25	6.58	3.22	
**L**^**4**^	9.58(1)	13.35(2)	19.76(3)		9.58	6.77	3.41	
**L**^**5**^	9.47(1)	15.15(2)	18.31(2)		9.47	5.68	3.16	
**L**^**6**^	9.43(1)	15.65(1)	18.91(1)		9.43	6.22	3.26	
**L**^**7**^	9.94(1)	16.93(2)	20.21(3)		9.94	6.99	3.28	
**L**^**8**^	9.96(1)	17.34 (1)	22.07(3)	25.39(3)	9.96	7.38	4.73	3.32
**L**^**9**^	9.70 (1)	17.26 (2)	22.41(4)	25.58(4)	9.70	7.56	5.15	3.17
**L**^**10**^	9.66 (1)	17.23 (1)	22.74(2)	25.96(3)	9.66	7.57	5.51	3.22
**L**^**11**^	9.55(1)	15.93 (2)	19.33(2)		9.55	6.38	3.40	
**L**^**12**^	9.63 (1)	16.37 (2)	19.60(2)		9.63	6.74	3.23	
**Triapine**	10.64 (1)	14.12 (1)			10.64	3.48		
**Triapine**	10.86 (1)^[^^45^^]^	14.65 (1)^[^^45^^]^			10.86	3.79		

^a^ Solvent MeOH/H_2_O 80/20 by weight

I = 0.1 M KCl, T = 25.0°C. The reported errors on log*β* are given as 1σ.

The UV/Vis spectrophotometric titrations revealed characteristic spectral changes in the 240–450 nm wavelength range. All studied ligands displayed intense absorption bands in this range, which were dependent on the protonated state of the molecule. The bands with a maximum absorption λ_max_ ≈ 380 nm-404 nm were assigned to the n→π* transitions of the pyridine ring. The less intense bands at ≈ 290 nm originated mainly from the π→π* transitions of the azomethine chromophore [45–47]. During the first deprotonation step ([H_3_L]^2+^ → [H_2_L]^+^, we observed a blueshift and a decrease in the intensity of the absorption maximum in the visible region. The next two deprotonation steps were accompanied by a redshift and an increase in intensity (Fig 1B and S1 Fig). Following the deprotonation of the N^2^-H group, the negative charge is transferred mainly to the S atom via the thione-thiol tautomeric equilibrium.

The proton dissociation constants and the spectra of the individual ligand species (Table 1) were calculated based on the deconvolution of the pH-dependent UV-Vis spectra. The obtained log*K*_a_ values are in a reasonably good agreement with previously reported thiosemicarbazones ligands [45–47]. The concentration distribution curves, together with the electronic spectra as a function of pH, are reported in the Supporting Information (S1 Fig).

For the studied ligands, the first protonation constant corresponded to the protonation of the hydrazanic = N-NH group. A decreased value, about one order of magnitude lower than in the other reported *N*-pyridyl thiosemicarbazones ligands (log*K*∼ 10.2–11), can most probably be attributed to the substitution of the piperazine ring by an aromatic ring (L^1^-L^7^), pyridine (L^8^-L^10^) or the pyrimidine and pyrazine moiety in L^11^ and L^12^, respectively. This effect is the result of the electron-withdrawing effect of these substituents. On other hand, the hydrogen bond between the pyridyl nitrogen and the = N-NH hydrazanic moiety is most probably responsible for the marked differences in the value of log*K*. The next protonation constant, log*K* with a value of 5.7–7.2 was assigned to the piperazine moiety. The obtained values of log*K* of the piperazine functional group of the studied ligands are comparable to the reported values [48–50] or log*K* values determined for the other TSC-based hybrids, mPip-FTSC and mPip-dm-FTSC (log*K* 7.28) [46]. The log*K* with value 3.1–3.4 can presumable be attributed to protonation of the pyridinium nitrogen. These values are in good agreement with the most popular TSC as is Triapine and other published *α-N*- pyridyl thiosemicarbazones [45,51]. For ligands L^8^-L^10^ were observed additional log*K* with value 4.73; 5.15 and 5.51 respectively. These values most probably corresponded to protonation of additional pyridinium unit, which is in agreement with the tabulated log*K* values of pyridine (5.23) [52].

The next acidic log*K*_*s*_ occurred well below pH 2.5 and the constants corresponding to these processes were not determined under the experimental conditions used in this study.

It should be noted that all of the measured log*K*s are macroscopic constants and that further constants cannot be ascribed to the protonation of either of the donor groups without detailed NMR titrations of the ligand [53]. However, taking into account the solvent conditions (*vide supra*) and comparing the observed data with those of a series of previously investigated ligands [45,46], the basicity of the substituents included in the studied ligand most probably follows the trend–hydrazine N^2^H group > piperazine > additional function group (e.g. pyridine, pyrimidine or pyrazine) ≈ pyridine.

For the studied ligands, the neutral uncharged form dominated at a physiological pH of 7–8.5, thus enabling a facile passage across the cell membranes. This would explain, at least in part, the high biological activity of these chelators by mobilizing intracellular Fe; preventing Fe uptake from the serum Fe transport protein, transferrin (Tf) and also inhibiting cellular proliferation [20]. The protonated form (Fig 3) became dominant below pH 5, while the deprotonated form was only important above pH 11 (Fig 3 and S1 Fig). Hence, if these agents are ever given as drugs via the oral route, the low pH of the stomach (pH 1–2) would prevent the absorption of the drug as the molecule would be charged [54]. More facile absorption would occur in the small intestine where the higher pH (pH 5–7) would result in a neutral ligand and a greater uptake [54].

#### Ability to chelate Cu(II) and Fe(III)

**Fig 4:**
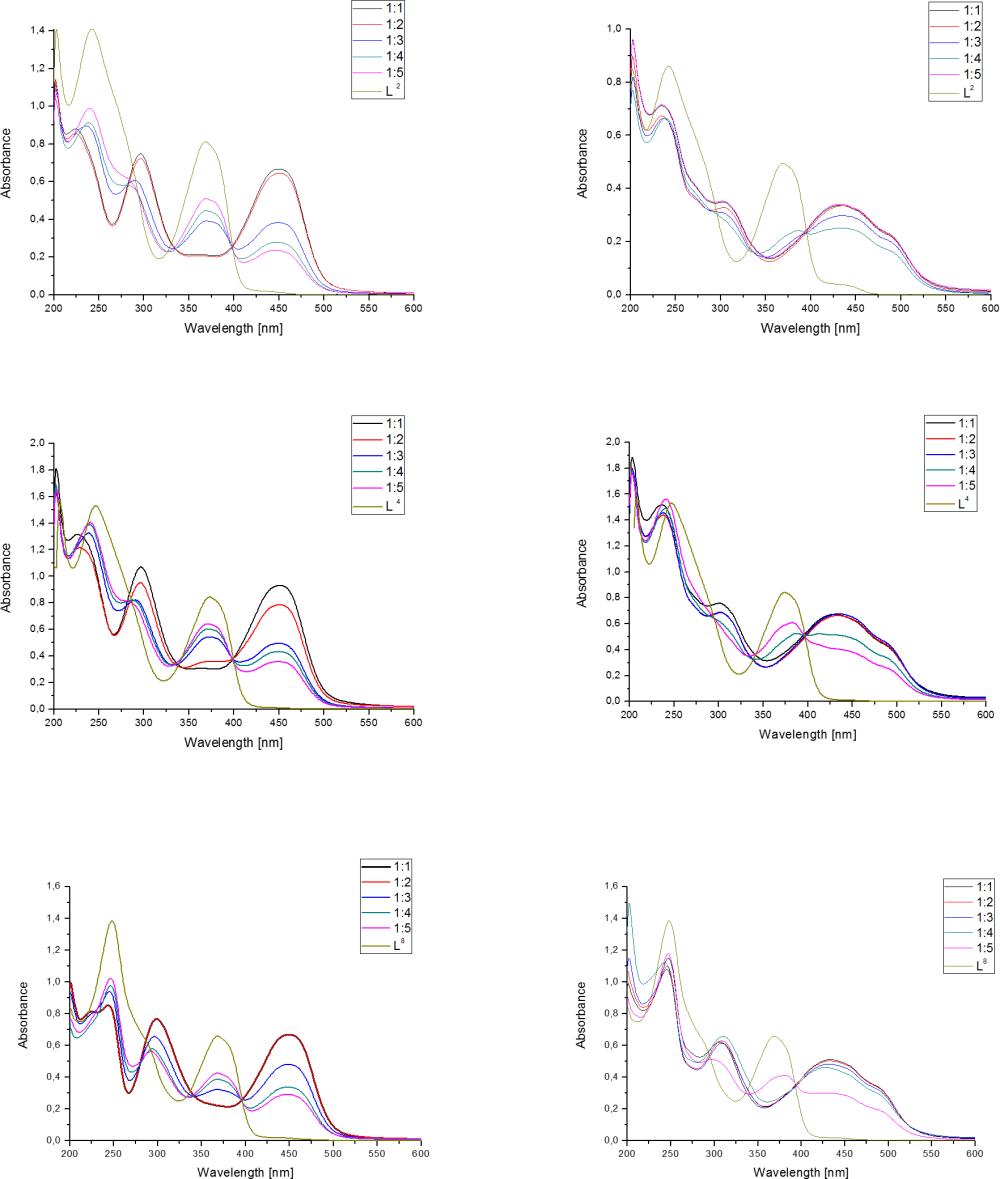
Electronic absorption spectra of the Cu(II) and Fe(III)-L^2^; L^4^; L^8^ system recorded at various metal to ligand ratios. I = 0.1 M (KCl) in 80% (w/w) MeOH/H_2_O; T = 25.0°C; [L] = 5x10^-5^M.

To evaluate the complex formation ability for the studied Triapine-derivative ligands, we performed the spectroscopic titration of the solutions of all the L^1^-L^12^ compounds with copper and iron ions. We titrated the studied ligands with the above-mentioned metal ions to generate their metal-complexes *in situ* at the following metal to ligand ratios 1:1–1:5. The isosbestic curves for the selected ligands are presented in Fig 4.

In general, the addition of a metal ion to the ligand solution caused distinguishable changes in the visible region of the ligand spectra. This behavior suggests an instantaneous complex formation in the solution from the reaction of each of the studied ligands with the metal ions used. All the ligands displayed the characteristic intense transitions in the range 400–500 nm for Cu(II) and 380–510 nm for Fe(III) that spanned into the visible region. Fig 4 shows the electronic spectral change that was observed in the MeOH/H_2_O 80/20 w/w solution when Cu(II) or Fe(III) was added to the selected ligands L^2^, L^4^ and L^8^. In the absence of metal ions, the light yellow solution showed intensive absorption bands at λ_max_ ≈ 250 nm, λ_max_ ≈ 290 nm and λ_max_ ≈ 380 nm—404 nm. As the Cu(II) or Fe(III) ion solution was added, the signals of the ligand alone decreased and those of the complexes increased in intensity at 430–450 nm with some isosbestic points at ≈ 337 nm and 395 nm (S1 Table). The presence of this band indicates a LMCT (*ligand to metal charge transfer*), which is typical of the Cu(II) L complex. This transition band corresponds to the S→Cu(II) and N_py_→Cu(II) transition. Similar ligand to metal charge transfer bands have also been observed in the complexes of other TSC ligands [45,46,51]. No d-d bands were observed due to the low concentration (~ 10^-5^M range) of the Cu(II) complexes in the solution. These bands should have a low intensity in the region of 600–700 nm.

In the studied ligands, the formation of mono complexes for Fe(III) resulted in a shoulder in the interval 450–620 nm. As metal-free ligands do not absorb in this region, these are most probably charge-transfer (CT) bands of the Fe-L complexes [46,51]. Additionally, we developed the characteristic CT bands λ_max_ ≈ 639 nm of the Fe(III)-L complexes in the wavelength of 580–680 nm, which was also detected in the other Fe(III)-TSC systems [46,51]. In conclusion, the analysis of the results showed that the presented ligands can act as effective copper or iron chelators.

### Anticancer activity

#### Cytotoxicity of novel Triapine analogs

All the newly synthesized compounds were evaluated against a panel of cancer and normal cell lines for their cytotoxic activity (Table 2). Among the cancer cells, we analyzed the cytotoxicity of the tested compounds against colon cancer with a normal (HCT116 p53^+/+^) and deleted TP53 suppressor gene (HCT116 p53^-/-^), which encodes the p53 protein. This was designed to allow the assayed activities to be compared with the literature data as a number of previous reports on the antiproliferative potency of TSCs presented results from these cells [21,41,42,55–58]. Moreover, colon cancers are among those that are particularly associated with a higher iron uptake and metabolism [59,60]. This makes them potentially vulnerable to treatments that target iron homeostasis. We also tested the cell lines of the brain tumor (U-251, Hs683) and breast cancer (MCF-7) cell lines. Breast cancers are among the most frequent cancers and their aggressiveness and treatment prognosis is strongly connected with iron metabolism [61–63]. Namely, it has been confirmed that the regulation pattern of the iron regulatory genes determined the metastasis potency and may be used as a predictive factor in the therapeutic strategy planning [62,64]. Glioblastomas, on the other hand, are one of the most dangerous cancers because no effective therapeutic regimes exist to treat them. The prognoses in these types of cancer are generally poor and the mean survival time does not exceed two years even under combination therapy [65,66]. Interestingly, those cancers also express an altered metabolism of iron and remain susceptible to treatments with iron chelators [67]. Both desferioxamine (DFO) and deferiprone have shown promising results in the U-251 cell line [68]. However, despite these encouraging promises, there are very few reports concerning the activity of TSCs in glioblastomas. Importantly, for the compounds to be useful as anti-cancer drugs, the selectivity between the tumor cells and normal, mortal cell types must be revealed. Therefore, we additionally tested the new compounds on the NHDF cell line. In general, all the examined analogs showed a significantly higher anti-proliferative activity than the reference Triapine. The most active compound was compound L^3^ with an IC_50_ value equal to 0.12 μM in the HCT116 p53^+/+^ cell line. The activity of this compound was very similar for all the cancer lines and fluctuated around 0.12–0.2 μM. This derivative also has a promising therapeutic index (208 in HCT116 p53^+/+^ see S2 Table) as its cytotoxicity against normal cell lines is relatively low. However, the glioma cell line Hs683 is an exception here as it has an IC_50_ with a ten-fold higher value for L^3^. In fact, the glioblastoma Hs683 exhibited resistance to almost all the tested analogs except for the L^9^ and L^10^ derivatives. Those two analogs were active against all the tested cancer cell lines. In general, ligands that are halogenated at the terminal aromatic ring exerted a higher activity than their unsubstituted non-halogenated counterparts (compare L^1^ vs. L^2^, L^3^). The compounds with diazine rings appeared to be less active than phenol (L^1^ vs. L^11^ and L^12^) and the pyridine ring produced a derivative that was even less active (L^8^). This may have been the result of the unfavorable pK*a* of the molecule and is in contrast with previous reports [21]. The trifluoromethyl substitution was very active against all the cancer cell lines that were tested. It also appeared to be less toxic against normal fibroblasts (L^6^, L^9^, L^10^).

**Table 2:** Anti-proliferative activity (IC_50_ values) of the novel Triapine analogs compared to Triapine in several tumor cell-types and normal human dermal fibroblast (NHDF) cells. Individual IC50 values IC50 < 1μM, IC50 1–10 μM, IC50 > 10 μM are coded by red, yellow and grey, respectively.

Name	IC_50_ [μM]					
	HCT116 p53^+/+^	HCT116 p53^-/-^	MCF-7	U-251	Hs683	NHDF
**L**^**1**^	1.524 ± 0.445	0.128 ± 0.012	0.532 ± 0.145	0.403 ± 0.090	1.436 ± 0.358	>25
**L**^**2**^	0.526 ± 0.077	0.152 ± 0.074	0.360 ± 0.111	0.887 ± 0.158	2.487 ± 0.512	>25
**L**^**3**^	0.120 ± 0.005	0.167 ± 0.018	0.204 ± 0.049	0.128 ± 0.012	1.483 ± 0.335	>25
**L**^**4**^	1.328 ± 0.209	0.185 ± 0.088	0.424 ± 0.149	0.649 ± 0.170	4.457 ± 0.954	>25
**L**^**5**^	1.450 ± 0.452	0.7461 ± 0.354	3.807 ± 1.023	2.569 ± 0.675	10.050 ± 2.643	>25
**L**^**6**^	0.139 ± 0.016	0.270 ± 0.007	0.470 ± 0.097	0.381 ± 0.042	1.894 ± 0.812	>25
**L**^**7**^	0.762 ± 0.238	1.133 ± 0.049	2.536 ± 0.400	3.084 ± 1.273	4.624 ± 1.031	>25
**L**^**8**^	1.900 ± 0.292	0.139 ± 0.020	1.120 ± 0.113	1.070 ± 0.280	1.162 ± 0.307	>25
**L**^**9**^	0.422 ± 0.113	0.123 ± 0.061	0.204 ± 0.024	0.277 ± 0.058	0.844 ± 0.238	>25
**L**^**10**^	0.170 ± 0.015	0.159 ± 0.011	0.258 ± 0.040	0.137 ± 0.016	0.195 ± 0.021	>25
**L**^**11**^	0.668 ± 0.045	0.650 ± 0.055	1.730 ± 0.388	1.268 ± 0.374	2.838 ± 0.581	>25
**L**^**12**^	0.435 ± 0.108	0.342 ± 0.042	2.218 ± 0.638	0.743 ± 0.109	2.595 ± 0.237	>25
**L**^**13**^**/3-AP**	1.121 ± 0.277	1.336 ± 0.338	2.328 ± 0.431	1.476 ± 0.558	1.763 ± 0.292	>25

The effect of the TP53 status on a cell’s susceptibility to the some of the Triapine analogs is interesting. The derivatives L^1^, L^4^, L^5^ and L^8^ were approximately 2–14 times more effective against HCT116 (p53^-/-^) than against the wild-type cells. In turn, this scheme was inverted for compound L^7^, which exhibited a reversed effect, as these compounds appeared to be slightly less active against the p53^-/-^ cell line. In addition to these observations, the colon cancer cells, breast and U-251 glioma appeared to be similarly susceptible to TSCs. A particularly interesting exception was the Hs683 cell line, which appeared to be relatively resistant to almost all of the compounds with a ten-fold higher mean IC_50_. This phenomenon is important when considering the similar origin of the U-251 and Hs683 lines (from a malignant glioblastoma tumor and from a glioma lesion of 75-year-old Caucasian male patient, respectively). The explanation probably lies in a different level of the basal iron homeostasis, e.g. originating from the different activity and concentration of the transferrin (Tf) receptors. It has previously been reported that Hs683 is significantly more resistant than U-251 to Tf-toxins such as the pokeweed antiviral protein, momordin or gelonin [69]. This suggests that in resistant cells, the cellular metabolism of iron can be restored more easily.

To summarize, the novel compounds demonstrated the greatest promise as anti-cancer agents with both a potent and selective anti-proliferative activity (Table 2). Accordingly, the structure-activity relationship reveals that the combination of the piperazine ring with Triapine allows potent and selective anticancer chelators that warrant further *in vivo* examination to be identified. In particular, the compounds L^9^ and L^10^, which have fluorine atoms, appeared to have the best activity against all of the cancer lines that were tested. For this reason, we selected L^9^ for a more thorough investigation of the molecular mechanisms of its activity.

#### Cell cycle analysis

**Fig 5:**
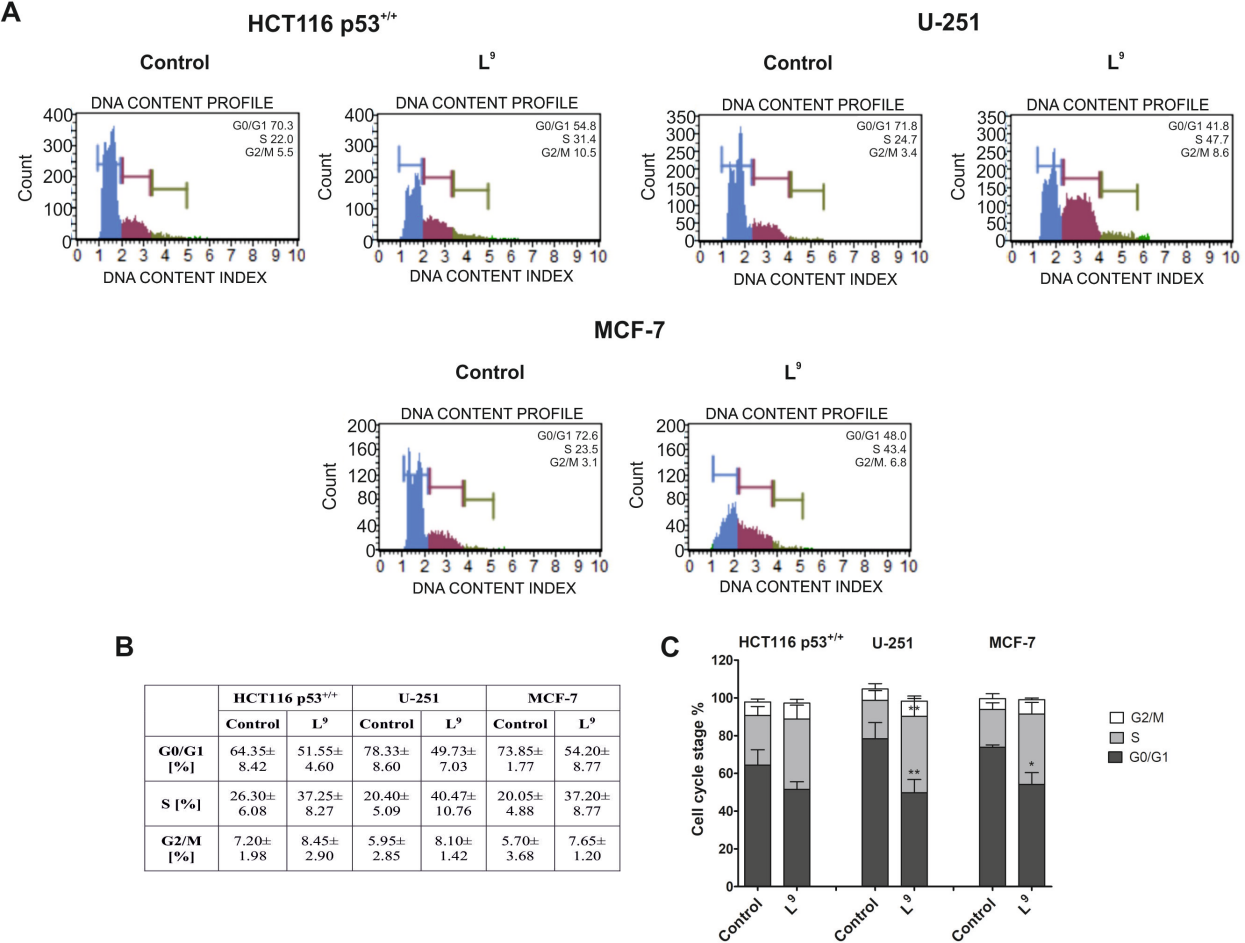
Influence of L^9^ on the regulation of the cell cycle in the HCT116 p53^+/+^, U-251 and MCF-7 cells. The histograms show the percentage of cells in the G0/G1, S and G2/M phases of the cell cycle for one of the experiments (A). The table shows the mean ± SD percentage of the cells in the G0/G1, S and G2/M phases of the cell cycle from three independent experiments (B). Data were analyzed using one-way ANOVA with Bonferroni’s post-hoc test: *p<0.05, **p<0.01, ***p<0.001 compared to the control (**C**).

The results presented in Fig 5 illustrate the effects of L^9^, which is one of the most active TSCs of the current series, on the regulation of the cell cycle in the HCT116 p53^+/+^, U-251 and MCF-7 cell lines. In general, we observed a decrease in the percentage of cells in the G0/G1 phase 24 h after treatment with L^9^ in all the cell lines. This effect was especially strong in the U-251 cells, in which L^9^ decreased the cell count in the G0/G1 phase to 50% compared to the untreated cells (78%) (Fig 5B). Additionally, the L^9^ compound induced an increase in the percentage of cells in the S phase in all of the tested cell lines. The strongest effect was observed for U-251 (41%). Moreover, 24 h after treatment with L^9^, we did not observe any changes in the percentage of cells in the G2/M phase compared to the untreated control. These results suggest that the tested TSCs may induce the arrest of the cell cycle in the G1/S phase, thus contributing to the induction of cell death. This result is in good agreement with literature data for TSC [70,71] as well as iron chelator in general [72–74]].

#### Analysis of programmed cell death–annexin V-FITC assay

**Fig 6:**
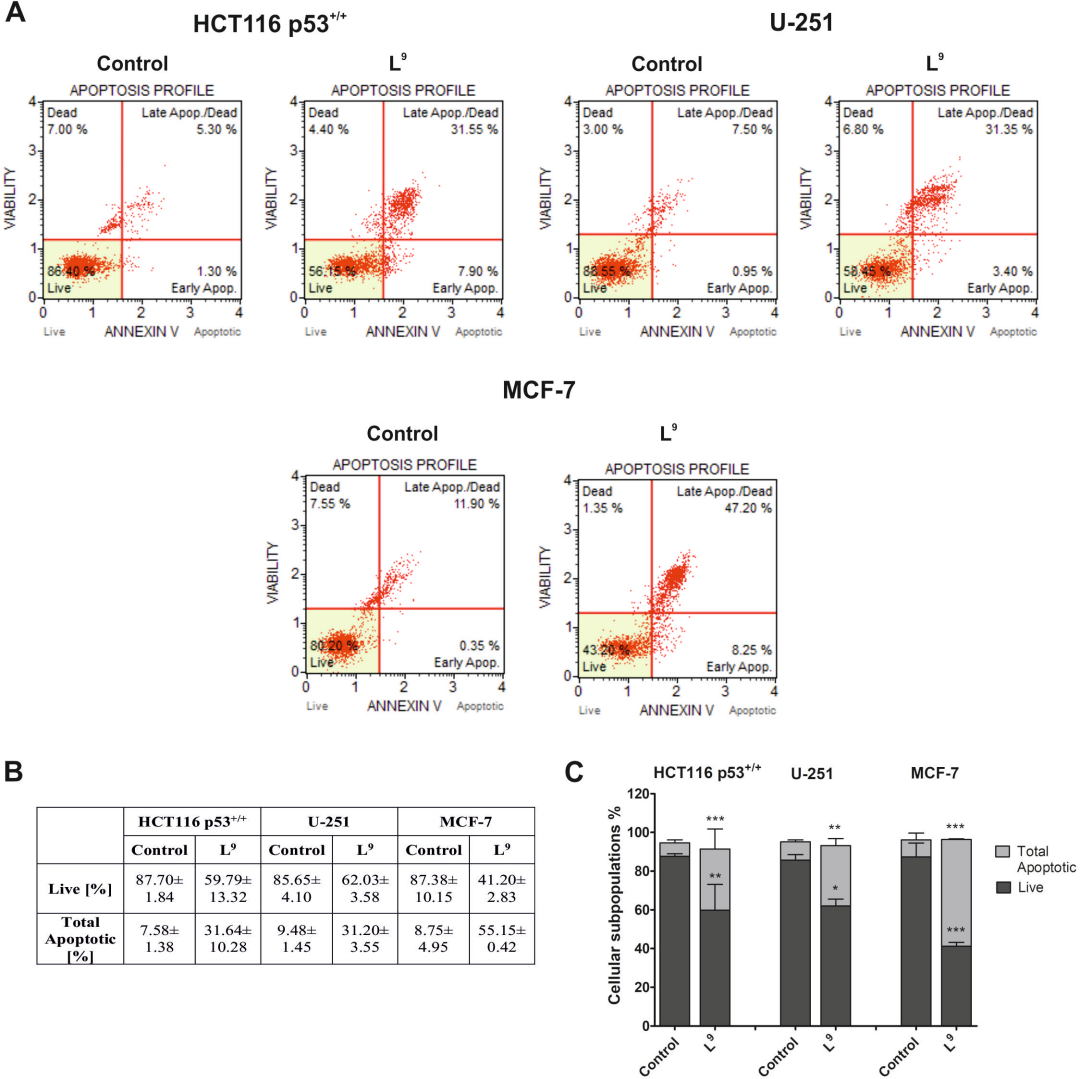
Evaluation of the induction of apoptosis in the HCT116 p53^+/+^, U-251 and MCF-7 cells 48 h after treatment with L^9^. The histograms show the percentage of early and late apoptosis for one of three independent experiments (**A**). The table shows the mean ± SD percentage of live, early and late apoptotic cells from three independent experiments (**B**). Data were analyzed using one-way ANOVA with Bonferroni’s post-hoc test: *p<0.05, **p<0.01, ***p<0.001 compared to the control (**C**).

The ability of L^9^ to induce apoptosis in the HCT116, U-251 and MCF-7 cells was determined using Annexin V-FITC staining. The results are presented in Fig 6. The largest percentage of apoptotic cells was observed in the MCF-7 cell line. In this case, we detected a six-fold increase in the number of dead cells (compared to the untreated cells). The total apoptotic value was 55% (Fig 6B). For HCT116 p53^+/+^, we also noticed a four-fold increase in the number of dead cells. The situation was different for the U-251 cell line, in which the percentage of total apoptotic cells was three times higher in L^9^-treated cells than the control. All observed changes are statistically significant.

#### Western blot analysis of the cell cycle and cell death proteins

**Fig 7:**
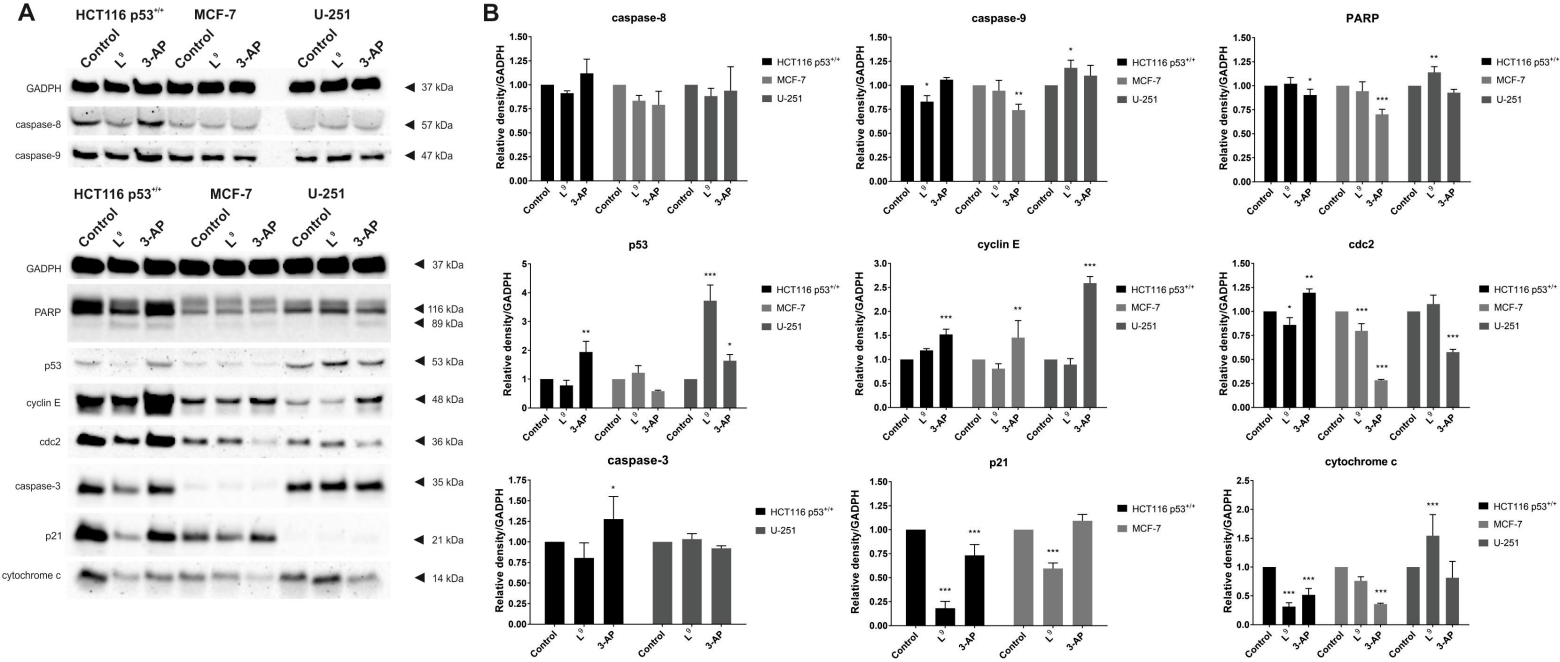
The effect of L^9^ on the expression of the proteins: GADPH, PARP, caspase-3, 8, 9, p53, cyclin E, cdc2, p21, and cytochrome c, in the HCT116 p53^+/+^, U-251 and MCF-7 cells. **(A).** Densitometric analyses of western blot images. Expression level signals are relative to GADPH expression. Data were analyzed using one-way ANOVA with Bonferroni’s post-hoc test: *p<0.05, **p<0.01, ***p<0.001 compared to the control (**B**).

Alterations in the iron level are associated with the progression of the cell cycle. Due to their ability to bind with cellular iron, iron chelators may affect the expression of many proteins that are responsible for controlling the cell cycle. The most important regulators of the cell cycle are cyclins, cyclin-dependent kinases (cdks), p53 and cyclin-dependent kinase inhibitors such as p21 [73]. The cdks are dependent on the cyclins to modulate their phosphorylation activity, and therefore the activity of the cyclin-cdk complexes are affected by the cyclin-dependent kinase inhibitors. The progression through the G1 phase and then transition to the S phase of the cell cycle are controlled in part by the activation of the cyclin D1/cdk4 and cyclin E/cdk2 complexes [74]. Additionally, the activity of cyclin D1 is associated with the p21 protein, which plays a crucial role in triggering various effects on cell cycle regulation. Thus, a decrease in the expression of the p21^CIP1/WAF1^ protein may lead to the arrest of the cell cycle in the G1/S phase since this protein can stabilize the cyclin D1-cdk complexes [75,76]. On the other hand, the effect of iron chelation may up-regulate p21^CIP1/WAF1^, which may induce the signaling pathways, thus leading to apoptosis [74,77]. In turn, the cdc2 protein, which is the catalytic subunit that complexes cyclin A, B, is responsible for the transition into the G2/M phase of the cell cycle [74,77]. With this in mind, we evaluated the impact of L^9^, as well as reference 3-AP, on the expression of cyclin E, p21 and the cdc2 proteins in HCT116 p53^+/+^, U-251 and MCF-7 cells. As is presented in Fig 7, we observed various patterns of the expression of the cell cycle proteins in the investigated cell lines. Treatment with L^9^ led to a slight increase in the expression of cyclin E in the HCT116 cells. Reversely, we observed a slight down-regulation of this protein in the other cell lines. A reference compound (3-AP) caused overexpression od cyclin E in all testes cell lines. Moreover, the western blot analysis revealed the influence of L^9^, especially, by the significant decrease of the expression of p21 protein in HCT116 cells. On the other hand, after treatment with L^9^, we observed the considerable down-regulation of p21 in the MCF-7 cells. We observed the similar pattern in changes in the expression of the cdc2 protein. Investigated compound influenced on the slight down-regulation of the cdc2 in the HCT116, and MCF-7 cell lines. In the case of 3-AP this effect was much stronger (MCF-7). This suggests that the TSCs have no influence on the entry of cells into the mitosis phase of the cell cycle. The obtained results indicate that the novel analogs of Triapine induced the arrest of the cell cycle in the G1/S phase and thus triggered apoptosis. Typically, this effect is connected with the activation of caspases–a family of endoproteases that participates in triggering the cellular response to damage. Caspase-8 is the initiator that participates in the extrinsic apoptosis pathway. Its activation comes via dimerization and leads to the initiation of the executioner caspases (-3, -6, -7) or activates the intrinsic pathway of apoptosis. The second type of cell death is also called mitochondrial apoptosis and is connected with the release of cytochrome c into the cytosol [78,79]. Therefore, we examined the effect of a 24 h treatment with L^9^ on the regulation of cytochrome c, and caspase-3, 8, 9 proteins. Additionally we explored another proteins, which are involved in the apoptosis pathway: p53, and PARP. Similar to previous results, we observed some differences among the investigated cell lines, which may be associated with various lengths of their cell cycle. The obtained results confirm that treatment with L^9^ induces the release of cytochrome c from the mitochondria into the cytosol in case of HCT116 cells. Additionally, we observed a slight down-regulation of cytochrome c in and MCF-7. For 3-AP we detected release of cytochrome c in all tested cell lines. To further elucidate the mechanism, we evaluated the influence of L^9^ on the extrinsic pathway of caspases activation.

In the case of caspase-3 we did not detect any significant changes in its expression for HCT116, and U-251 cell lines. It is commonly known that MCF-7 cell line do not express caspase-3 [80], and thus we investigate another caspase proteins. In general we observed the same pattern for caspase-8. In the case of caspase-9 we detected slight down-regulation for HCT116, and no influence on the MCF-7 cell line. A small up-regulation occurred for glioma cells. Results of caspases expression may indicate another pathway of the apoptosis triggering. Another investigated protein was p53, which is involved in many proliferating, and survival signals in cell. We detected interesting dependence in glioma cells, for which the upregulation of p53 was noticed. However it should be highlighted that U-251 cells are mutants with R273H missense mutation of p53. This resulted in normally expressed protein, that is able to cross the nucleus and interact with DNA [81,82]. Although overexpression of mutp53 in U-251 cells do not correlate with apoptosis. With this in mind increased level of cytochrome c and caspase-9 may suggest p53-independent apoptosis pathway. In colon and breast cancer influence of p53 protein was unnoticeable. Very popular protein for detection of cell death is PARP. Analysis of the lines on the gel indicated PARP cleavage for HCT116. This was also observed for glioma cell, but just for 3-AP. Summarizing, our hypothesis suggests influence of the cytochrome c (HCT116, MCF-7), and PARP (HCT116) on the apoptotic pathway.

## Conclusions

In the current study, a new class of TSCs, piperazinylogs of Triapine, was designed to fulfill the di-substitution pattern at the TSCs N4 position, which is a crucial prerequisite for the high activity of the previously obtained TSC compounds–DpC and Dp44mT. We tested the important physicochemical characteristics of the novel compounds L^1^-L^12^. The studied ligands are neutral at physiological pH, which allows them to permeate cell membranes and bind cellular Fe pools more readily than less lipid-soluble ligands, e.g. DFO.

The selectivity and anti-cancer activity of the novel TSCs were examined in a variety of cancer cell types. In general, the novel compounds demonstrated the greatest promise as anti-cancer agents with both a potent and selective anti-proliferative activity (Table 2). Accordingly, structure-activity relationship studies revealed that the combination of the piperazine ring with Triapine allows potent and selective anticancer chelators that warrant further *in vivo* examination to be identified. In particular, compounds L^6^ and L^10^ with a fluorine atom within the piperazine fragment appeared to enhance the activity and selectivity of the new analogs.

Significantly, this study proved the importance of the di-substitution pattern of the amine N4 function, thus identifying new potent and selective anticancer chelators that warrant further *in vivo* examination.

## Experimental section

### Chemistry

Microwave reactions were carried out in a Discover® BenchMate^TM^ (CEM) microwave equipped with 10 mL vessels. Melting point measurements were determined in a Stanford Research Systems OptiMelt (MPA 100). ^1^H and ^13^C NMR spectra were recorded on a Bruker Ascend 500 MHz spectrometer at frequencies of 500 MHz and 126 MHz and a Bruker Avance III 400 MHz FT-NMR spectrometer at frequencies of 400 MHz and 101 MHz using DMSO-*d*_*6*_ as the solvent and TMS as the internal standard. The NMR solvents were purchased from ACROS Organics. The chemical shifts (*δ*) are given in ppm and the coupling constants (*J*) values are reported in hertz (Hz). The spin multiplicities are described as s (singlet), d (doublet), dd (double of doublets), t (triplet), q (quartet) and m (multiplet). All evaporations were performed on a rotary evaporator under diminished pressure at 60°C. All reagents and solvents were purchased from ACROS Organics, Asta-Tech, Maybridge, Santa Cruz Biotechnology and Sigma-Aldrich and were used without further purification.

The purity of all compounds were tested using the HPLC/MS method. The HPLC–MS analyses were performed on Varian model 920 liquid chromatograph equipped with the Varian 900-LC model autosampler, the gradient pump, the Varian Pro Star 510 model column oven, the Varian 380-LC model evaporative light scattering detection (ELSD) detector. This was coupled with Varian 500-MS IT. HRMS were determined with high resolution mass spectrometer Waters LCT Premier XE with electrospray ionisation (ESI).

#### General procedure for the synthesis of thiosemicarbazides

The mixtures of (1,1'-thiocarbonyl) bis-1H-imidazole (5 mmol) and a suitable derivative of piperazine (5 mmol) in methylene chloride (25 mL) were stirred for 24 h at room temperature. The solutions were extracted with distilled water three times and the organic phases were dried over anhydrous magnesium sulfate, filtered and then evaporated on a rotary evaporator. The obtained derivatives of thioketone were added to a solution of 5 mmol of hydrazine hydrate in 25 mL of ethanol at room temperature. The reaction mixture was refluxed for 2 h and cooled to obtain a precipitate, which was collected via filtration. The final thiosemicarbazides were crystallized from methanol.

*4-phenylpiperazine-1-carbothiohydrazide* (**K**^**1**^)

White powder; yield 69%; mp: 173–174; ^1^H-NMR (400 MHz, *d*_*6*_-DMSO, ppm): δ 3.15 (s, 4H, CH_2_), 3.87 (s, 4H, CH_2_), 6.80 (t, 1H; *J* = 7.1 Hz), 6.95 (d, 2H; *J* = 8.0 Hz), 7.22 (t, 2H; *J* = 7.7 Hz), 9.17 (s, 1H, NH). ^13^C-NMR (126 MHz, *d*_*6*_-DMSO, ppm): δ 48.3; 56.5; 116.0; 119.7; 129.4; 151.1; 183.0. MS (ESI^+^): *m/z* calculated for C_11_H_16_N_4_S: 236.11, found: 237.64 [M+H]^+^, 276.05 [M+K]^+^.

*4-(4-fluorophenyl)piperazine-1-carbothiohydrazide* (**K**^**2**^)

Light pink powder; yield 97%; mp: 180–181; ^1^H-NMR (400 MHz, *d*_*6*_-DMSO, ppm): δ 3.09 (m, 4H, CH_2_), 3.87 (m, 4H, CH_2_), 4.77 (s, 2H, NH_2_), 6.97 (m, 2H, CH), 7.05 (m, 2H, CH), 9.19 (s, 1H, NH). ^13^C-NMR (101 MHz, *d*_*6*_-DMSO, ppm): δ 40.3; 49.1; 115.9; 117.9; 148.0; 157.8; 183.0. MS (ESI^+^): *m/z* calculated for C_11_H_15_FN_4_S: 254.10, found: 276.30 [M+Na]^+^.

*4-(4-chlorophenyl)piperazine-1-carbothiohydrazide* (**K**^**3**^)

White powder; yield 90%; mp: 195–196; ^1^H-NMR (500 MHz, *d*_*6*_-DMSO, ppm): δ 3.16 (t, 4H, *J* = 5.2 Hz), 3.86 (t, 4H, *J* = 5.2 Hz), 6.95 (m, 2H, CH), 7.24 (m, 2H, CH), 9.18 (s, 1H, NH). ^13^C-NMR (126 MHz, *d*_*6*_-DMSO, ppm): δ 47.3; 48.0; 117.3; 123.0; 129.1; 149.8; 182.9. MS (ESI^+^): *m/z* calculated for C_11_H_15_ClN_4_S: 270.07, found: 270.35 [M]^+^.

*4-(4-methoxyphenyl)piperazine-1-carbothiohydrazide* (**K**^**4**^)

White powder; yield 81%; mp: 194–195; ^1^H-NMR (400 MHz, *d*_*6*_-DMSO, ppm): δ 3.00 (m, 4H, CH_2_), 3.68 (s, 3H, CH_3_), 3.86 (m, 4H, CH_2_), 4.76 (s, 2H, NH_2_), 6.83 (m, 2H, CH), 6.92 (m, 2H, CH), 9.17 (s, 1H, NH).^13^C-NMR (101 MHz, *d*_*6*_-DMSO, ppm): δ 19.0; 49.9; 56.5; 114.8; 118.2; 145.4; 153.7; 183.1. MS (ESI^+^): *m/z* calculated for C_12_H_18_N_4_OS: 266.12, found: 288.02 [M+Na]^+^.

*4-(4-cyanophenyl)piperazine-1-carbothiohydrazide* (**K**^**5**^)

White powder; yield 86%; mp: 179–180; ^1^H-NMR (400 MHz, *d*_*6*_-DMSO, ppm): δ 3.42 (m, 4H, CH_2_), 3.88 (m, 4H, CH_2_), 4.76 (s, 2H, NH_2_), 6.99 (m, 2H, CH), 7.59 (m, 2H, CH), 9.15 (s, 1H, NH). ^13^C-NMR (126 MHz, *d*_*6*_-DMSO, ppm): δ 40.6; 48.8; 98.6; 114.3; 120.5; 133.8; 153.1; 182.9. MS (ESI^+^): *m/z* calculated for C_12_H_15_N_5_S: 261.10, found: 284.75 [M+Na]^+^.

*4-[2-nitro-4-(trifluoromethyl)phenyl]piperazine-1-carbothiohydrazide* (**K**^**6**^)

Light orange crystals; yield 75%; mp: 167–168; ^1^H-NMR (500 MHz, *d*_*6*_-DMSO, ppm): δ 3.26 (m, 4H, CH_2_), 3.88 (m, 4H, CH_2_), 4.76 (s, 2H, NH_2_), 7.44 (d, 1H; *J* = 8.8 Hz), 7.86 (dd, 1H; *J*_*1*_ = 9.0 Hz, *J*_*2*_ = 2.3 Hz), 8.17 (dd, 1H; *J*_*1*_ = 2.3 Hz, *J*_*2*_ = 0.9 Hz), 9.15 (s, 1H, NH). ^13^C-NMR (126 MHz, *d*_*6*_-DMSO, ppm): δ 46.9; 49.4; 119.7; 121.2; 123.0; 124.4; 130.5; 139.1; 147.5; 183.0. MS (ESI^+^): *m/z* calculated for C_12_H_14_F_3_N_5_O_2_S: 349.08, found: 349.05 [M]^+^.

*tert-butyl 4-(hydrazinylcarbonothioyl)piperazine-1-carboxylate* (**K**^**7**^)

White powder; yield 70%; mp: 165–167; ^1^H-NMR (500 MHz, *d*_*6*_-DMSO, ppm): δ 1.41 (s, 9H, CH_3_), 3.32 (m, 4H, CH_2_), 3.71 (m, 4H, CH_2_), 4.72 (s, 2H, NH_2_), 9.11 (s, 1H, NH). ^13^C-NMR (126 MHz, *d*_*6*_-DMSO, ppm): δ 19.0; 28.5; 47.4; 49.0; 79.6; 154.3; 183.1. MS (ESI^+^): *m/z* calculated for C_10_H_20_N_4_O_2_S: 260.13, found: 283.77 [M+Na]^+^.

*4-(pyridin-2-yl)piperazine-1-carbothiohydrazide* (**K**^**8**^)

White powder; yield 98%; mp: 172–173; ^1^H-NMR (400 MHz, *d*_*6*_-DMSO, ppm): δ 3.18 (s, 4H, CH_2_), 3.85 (m, 4H, CH_2_), 4.77 (s, 2H, NH_2_), 6.83 (m, 1H, CH), 7.56 (m, 1H, CH), 8.13 (m, 1H, CH), 9.13 (s, 1H, NH). ^13^C-NMR (101 MHz, *d*_*6*_-DMSO, ppm): δ 39.6; 51.0; 107.6; 113.7; 138.0; 148.0; 159.1; 183.0. MS (ESI^+^): *m/z* calculated for C_10_H_15_N_5_S: 237.10, found: 237.65 [M]^+^.

*4-[5-(trifluoromethyl)pyridin-2-yl]piperazine-1-carbothiohydrazide* (**K**^**9**^)

White powder; yield 70%; mp: 206–207; ^1^H-NMR (400 MHz, *d*_*6*_-DMSO, ppm): δ 3.70 (s, 4H, CH_2_), 3.87 (s, 4H, CH_2_), 4.76 (s, 2H, NH_2_), 6.93 (d, 1H, *J* = 9.1 Hz), 7.82 (s, 1H, CH), 8.42 (s, 1H, CH), 9.14 (s, 1H, NH). ^13^C-NMR (101 MHz, *d*_*6*_-DMSO, ppm): δ 43.8; 46.9; 49.1; 106.7; 134.9; 145.6; 145.7; 160.3; 183.0. MS (ESI^+^): *m/z* calculated for C_11_H_14_F_3_N_5_S: 305.09, found: 305.25 [M]^+^.

*4-[3-chloro-5-(trifluoromethyl)pyridin-2-yl]piperazine-1-carbothiohydrazide* (**K**^**10**^)

White powder; yield 85%; mp: 191–192; ^1^H-NMR (500 MHz, *d*_*6*_-DMSO, ppm): δ 3.51 (m, 4H, CH_2_), 3.88 (m, 4H, CH_2_), 4.83 (s, 2H, NH_2_), 8.20 (d, 1H; *J* = 2.1 Hz), 8.56 (s, 1H, CH), 9.18 (s, 1H, NH). ^13^C-NMR (126 MHz, *d*_*6*_-DMSO, ppm): δ 47.3; 48.1; 120.2; 122.8; 125.0; 136.8; 143.5; 159.7; 183.2. MS (ESI^+^): *m/z* calculated for C_11_H_13_ClF_3_N_5_S: 339.05, found: 340.28 [M+H]^+^.

*4-(pyrimidin-2-yl)piperazine-1-carbothiohydrazide* (**K**^**11**^)

White powder; yield 92%; mp: 208–209; ^1^H-NMR (500 MHz, *d*_*6*_-DMSO, ppm): δ 3.75 (m, 4H, CH_2_), 3.83 (m, 4H, CH_2_), 4.76 (s, 2H, NH_2_), 6.66 (t, 1H, *J* = 4.7 Hz), 8.38 (d, 2H, *J* = 4.7 Hz), 9.13 (s, 1H, NH). ^13^C-NMR (126 MHz, *d*_*6*_-DMSO, ppm): δ 43.2; 47.3; 110.9; 158.4; 161.5; 183.0. MS (ESI^+^): *m/z* calculated for C_9_H_14_N_6_S: 238.10, found: 238.35 [M]^+^.

*4-(pyrazin-2-yl)piperazine-1-carbothiohydrazide* (**K**^**12**^)

Light pink crystals; yield 88%; mp: 177–178; ^1^H-NMR (500 MHz, *d*_*6*_-DMSO, ppm): δ 3.61 (m, 4H, CH_2_), 3.87 (m, 4H, CH_2_), 4.77 (s, 2H, NH_2_), 7.86 (d, 1H; *J* = 2.7 Hz), 8.09 (dd, 1H; *J*_*1*_ = 2.7 Hz, *J*_*2*_ = 1.5 Hz), 8.31 (d, 1H, *J* = 1.5 Hz), 9.16 (s, 1H, NH). ^13^C-NMR (126 MHz, *d*_*6*_-DMSO, ppm): δ 43.7; 47.0; 131.7; 133.0; 141.9; 154.7; 182.9. MS (ESI^+^): *m/z* calculated for C_9_H_14_N_6_S: 238.10, found: 238.57 [M+H]^+^.

#### General procedure for synthesis of thiosemicarbazones

We added two drops of glacial acetic acid as a catalyst to the mixtures of thiosemicarbazides (0.5 mmol) and 3-aminopyridine-2-carboxaldehyde (0.5 mmol) in ethanol (5 mL). The glass tubes were sealed and placed into a microwave reactor at 83 ºC for 20 minutes (the reactor power did not exceed 50 W). The obtained thiosemicarbazones were crystallized from methanol.

*N’-[(3-aminopyridin-2-yl)methylidene]-4-phenylpiperazine-1-carbothiohydrazide* (**L**^**1**^)

Light yellow crystals; yield 83%; mp: 189–190; ^1^H-NMR (400 MHz, *d*_*6*_-DMSO, ppm): δ 3.26 (s, 4H, CH_2_), 4.09 (s, 4H, CH_2_), 6.82 (t, 1H; *J* = 7.3 Hz), 6.99 (d, 2H; *J* = 8.2 Hz), 7.10 (m, 2H, CH), 7.18 (s, 2H, NH_2_), 7.25 (t, 2H; *J* = 7.8 Hz), 7.84 (dd, 1H; *J*_*1*_ = 4.0 Hz, *J*_*2*_ = 1.6 Hz), 8.53 (s, 1H, CH), 11.44 (s, 1H, NH). ^13^C-NMR (126 MHz, *d*_*6*_-DMSO, ppm): δ 48.3; 48.6; 116.0; 119.6; 122.4; 124.5; 129.5; 134.2; 137.2; 144.2; 149.5; 150.9; 180.0. HRMS (ESI): *m/z* calculated for C_17_H_21_N_6_S: 341.1548, found: 341.1547 [M+H]^+^.

*N’-[(3-aminopyridin-2-yl)methylidene]-4-(4-fluorophenyl)piperazine-1-carbothiohydrazide* (**L**^**2**^)

Yellow crystals; yield 70%; mp: 210–211; ^1^H-NMR (400 MHz, *d*_*6*_-DMSO, ppm): δ 3.18 (s, 4H, CH_2_), 4.08 (s, 4H, CH_2_), 6.96–7.03 (m, 2H, CH), 7.04–7.13 (m, 4H, CH), 7.17 (s, 2H, NH_2_), 7.84 (d, 1H; *J* = 3.9 Hz), 8.53 (s, 1H, CH), 11.44 (s, 1H, NH). ^13^C-NMR (126 MHz, *d*_*6*_-DMSO, ppm): δ 48.6; 49.3; 115.8; 117.9; 122.4; 124.5; 134.2; 137.2; 144.2; 147.8; 149.5; 155.8; 157.6; 180.1. HRMS (ESI): *m/z* calculated for C_17_H_20_FN_6_S: 359.1454, found: 359.1466 [M+H]^+^.

*N’-[(3-aminopyridin-2-yl)methylidene]-4-(4-chlorophenyl)piperazine-1-carbothiohydrazide* (**L**^**3**^)

Yellow powder; yield 83%; mp: 206–207; ^1^H-NMR (500 MHz, *d*_*6*_-DMSO, ppm): δ 3.27 (t, 4H, *J* = 5.2 Hz), 4.08 (t, 4H, *J* = 5.3 Hz), 6.99 (m, 2H, CH), 7.06–7.08 (dd, 1H; *J*_*1*_ = 8.3 Hz, *J*_*2*_ = 4.2 Hz), 7.09–7.12 (dd, 1H; *J*_*1*_ = 8.4 Hz, *J*_*2*_ = 1.6 Hz), 7.14–7.22 (s, 2H, NH_2_), 7.25–7.28 (m, 2H, CH), 7.84 (dd, 1H; *J*_*1*_ = 4.2 Hz, *J*_*2*_ = 1.6 Hz), 8.53 (s, 1H, CH), 11.45 (s, 1H, NH). ^13^C-NMR (126 MHz, *d*_*6*_-DMSO, ppm): δ 48.0; 48.4; 117.3; 122.5; 123.1; 124.5; 129.1; 134.2; 137.2; 144.2; 149.5; 149.7; 180.1. HRMS (ESI): *m/z* calculated for C_17_H_20_ClN_6_S: 375.1159, found: 375.1155 [M+H]^+^.

*N’-[(3-aminopyridin-2-yl)methylidene]-4-(4-methoxyphenyl)piperazine-1-carbothiohydrazide* (**L**^**4**^)

Yellow powder; yield 59%; mp: 195–196; ^1^H-NMR (400 MHz, *d*_*6*_-DMSO, ppm): δ 3.11 (s, 4H, CH_2_), 3.70 (s, 3H, CH_3_), 4.07 (s, 4H, CH_2_), 6.85 (m, 2H, CH), 6.96 (m, 2H, CH), 7.09 (m, 2H, CH), 7.17 (s, 2H, NH_2_), 7.84 (dd, 1H; *J*_*1*_ = 4.1 Hz, *J*_*2*_ = 1.7 Hz), 8.52 (s, 1H, CH), 11.43 (s, 1H, NH). ^13^C-NMR (101 MHz, *d*_*6*_-DMSO, ppm): δ 48.8; 50.0; 55.7; 114.8; 118.3; 122.4; 124.5; 134.2; 137.1; 144.2; 145.3; 149.4; 153.7; 180.1. HRMS (ESI): *m/z* calculated for C_18_H_23_N_6_OS: 371.1654, found: 371.1645 [M+H]^+^.

*N’-[(3-aminopyridin-2-yl)methylidene]-4-(4-cyanophenyl)piperazine-1-carbothiohydrazide* (**L**^**5**^)

Light orange powder; yield 74%; mp: 209–210; ^1^H-NMR (400 MHz, *d*_*6*_-DMSO, ppm): δ 3.53 (s, 4H, CH_2_), 4.10 (s, 4H, CH_2_), 7.02 (d, 2H; *J* = 8.8 Hz), 7.09 (m, 2H, CH), 7.17 (s, 2H, NH_2_), 7.62 (d, 2H; *J* = 8.7 Hz), 7.84 (dd, 1H; *J*_*1*_ = 4.1 Hz, *J*_*2*_ = 1.6 Hz), 8.54 (s, 1H, CH), 11.42 (s, 1H, NH). ^13^C-NMR (126 MHz, *d*_*6*_-DMSO, ppm): δ 45.8; 47.9; 98.5; 114.0; 120.6; 122.5; 124.5; 133.8; 134.2; 137.2; 144.2; 149.6; 152.9; 179.9. HRMS (ESI): *m/z* calculated for C_18_H_20_N_7_S: 366.1501, found: 366.1503 [M+H]^+^.

*N’-[(3-aminopyridin-2-yl)methylidene]-4-[2-nitro-4-(trifluoromethyl)phenyl]piperazine-1-carbothiohydrazide* (**L**^**6**^)

Yellow powder; yield 75%; mp: 179–180; ^1^H-NMR (500 MHz, *d*_*6*_-DMSO, ppm): δ 3.38 (m, 4H, CH_2_), 4.09 (m, 4H, CH_2_), 7.07 (dd, 1H; *J*_*1*_ = 8.3 Hz, *J*_*2*_ = 4.2 Hz), 7.11 (dd, 1H; *J*_*1*_ = 8.4 Hz, *J*_*2*_ = 1.6 Hz), 7.13–7.21 (s, 2H, NH_2_), 7.47 (d, 1H; *J* = 8.9 Hz), 7.83–7.91 (m, 2H, CH), 8.20 (d, 1H, *J* = 2.7 Hz), 8.53 (s, 1H, CH), 11.42 (s, 1H, NH). ^13^C-NMR (126 MHz, *d*_*6*_-DMSO, ppm): δ 19.0; 47.9; 49.3; 56.5; 121.1; 122.5; 123.0; 124.4; 130.5; 134.2; 137.2; 138.9; 144.3; 147.5; 149.7; 180.1. HRMS (ESI): *m/z* calculated for C_18_H_19_F_3_N_7_O_2_S: 454.1293, found: 474.1269 [M+H]^+^.

*tert-butyl 4-({2-[(3-aminopyridin-2-yl)methylidene]hydrazinyl}carbonothioyl)piperazine-1-carboxylate* (**L**^**7**^)

Yellow powder; yield 20%; mp: 193–194; ^1^H-NMR (500 MHz, *d*_*6*_-DMSO, ppm): δ 1.43 (s, 9H, CH_3_), 3.44 (m, 4H, CH_2_), 3.93 (m, 4H, CH_2_), 7.05–7.12 (m, 2H, CH), 7.13–7.19 (s, 2H, NH_2_), 7.84 (dd, 1H; *J*_*1*_ = 4.2 Hz, *J*_*2*_ = 1.6 Hz), 8.51 (s, 1H, CH), 11.39 (s, 1H, NH). ^13^C-NMR (126 MHz, *d*_*6*_-DMSO, ppm): δ 28.5; 48.4; 79.7; 122.8; 124.5; 133.9; 136.9; 144.3; 149.2; 154.3; 180.2. HRMS (ESI): *m/z* calculated for C_16_H_25_N_6_O_2_S: 365.1760, found: 365.1769 [M+H]^+^.

*N’-[(3-aminopyridin-2-yl)methylidene]-4-(pyridin-2-yl)piperazine-1-carbothiohydrazide* (**L**^**8**^)

Yellow crystals; yield 38%; mp: 210–211; ^1^H-NMR (400 MHz, *d*_*6*_-DMSO, ppm): δ 3.64 (m, 4H, CH_2_), 4.07 (m, 4H, CH_2_), 6.68 (m, 1H, CH), 6.86 (d, 1H; *J* = 8.6 Hz), 7.09 (m, 2H, CH), 7.18 (s, 2H, NH_2_), 7.58 (m, 1H, CH), 7.84 (dd, 1H; *J*_*1*_ = 4.1 Hz, *J*_*2*_ = 1.6 Hz), 8.15 (d, 1H, *J* = 4.9 Hz), 8.54 (s, 1H, CH), 11.41 (s, 1H, NH). ^13^C-NMR (101 MHz, *d*_*6*_-DMSO, ppm): δ 44.4; 48.3; 107.5; 113.7; 122.5; 124.5; 134.2; 137.1; 138.1; 144.2; 148.0; 149.5; 159.0; 180.1. HRMS (ESI): *m/z* calculated for C_16_H_20_N_7_S: 342.1501, found: 342.1514 [M+H]^+^.

*N’-[(3-aminopyridin-2-yl)methylidene]-4-[5-(trifluoromethyl)pyridin-2-yl]piperazine-1-carbothiohydrazide* (**L**^**9**^)

Yellow crystals; yield 47%; mp: 232–233; ^1^H-NMR (400 MHz, *d*_*6*_-DMSO, ppm): δ 3.80 (s, 4H, CH_2_), 4.09 (s, 4H, CH_2_), 6.97 (d, 1H; *J* = 9.1 Hz), 7.08 (m, 2H, CH), 7.18 (s, 2H, NH_2_), 7.85 (m, 2H, CH), 8.46 (d, 1H, *J* = 2.5 Hz), 8.54 (s, 1H, CH), 11.42 (s, 1H, NH). ^13^C-NMR (101 MHz, *d*_*6*_-DMSO, ppm): δ 43.8; 47.9; 106.7; 113.8; 122.5; 124.5; 126.4; 134.2; 135.0; 137.1; 144.2; 145.7; 149.6; 160.3; 180.0. HRMS (ESI): *m/z* calculated for C_17_H_19_F_3_N_7_S: 410.1374, found: 410.1365 [M+H]^+^.

*4*.*1*.*2*.*10*. *N’-[(3-aminopyridin-2-yl)methylidene]-4-[3-chloro-5-(trifluoromethyl)pyridin-2-yl]piperazine-1-carbothiohydrazide* (**L**^**10**^)

Dark yellow powder; yield 64%; mp: 199–200; ^1^H-NMR (500 MHz, *d*_*6*_-DMSO, ppm): δ 3.63 (m, 4H, CH_2_), 4.10 (m, 4H, CH_2_), 7.05–7.09 (dd, 1H; *J*_*1*_ = 8.3 Hz, *J*_*2*_ = 4.2 Hz), 7.09–7.12 (dd, 1H; *J*_*1*_ = 8.3 Hz, *J*_*2*_ = 1.6 Hz), 7.14–7.21 (s, 2H, NH_2_), 7.84 (dd, 1H; *J*_*1*_ = 4.1 Hz, *J*_*2*_ = 1.6 Hz), 8.23 (d, 1H, *J* = 2.2 Hz), 8.53 (s, 1H, CH), 8.59 (dd, 1H; *J*_*1*_ = 2.3 Hz, *J*_*2*_ = 1.1 Hz), 11.44 (s, 1H, NH). ^13^C-NMR (126 MHz, *d*_*6*_-DMSO, ppm): δ 48.1; 48.3; 119.1; 120.0; 122.5; 124.5; 125.0; 134.2; 136.9; 137.2; 143.6; 144.2; 149.6; 159.5; 180.3. HRMS (ESI): *m/z* calculated for C_17_H_18_ClF_3_N_7_S: 444.0985, found: 464.0988 [M+H]^+^.

*N’-[(3-aminopyridin-2-yl)methylidene]-4-(pyrimidin-2-yl)piperazine-1-carbothiohydrazide* (**L**^**11**^)

Light yellow powder; yield 39%; mp: 198–199; ^1^H-NMR (500 MHz, *d*_*6*_-DMSO, ppm): δ 3.86 (m, 4H, CH_2_), 4.05 (m, 4H, CH_2_), 6.69 (t, 1H, *J* = 4.7 Hz), 7.07 (dd, 1H; *J*_*1*_ = 8.3 Hz, *J*_*2*_ = 4.2 Hz), 7.11 (dd, 1H; *J*_*1*_ = 8.3 Hz, *J*_*2*_ = 1.6 Hz), 7.13–7.22 (s, 2H, NH_2_), 7.84 (dd, 1H; *J*_*1*_ = 4.2 Hz, *J*_*2*_ = 1.6 Hz), 8.41 (d, 2H, *J* = 4.7 Hz), 8.53 (s, 1H, CH), 11.43 (s, 1H, NH). ^13^C-NMR (126 MHz, *d*_*6*_-DMSO, ppm): δ 43.2; 48.4; 111.0; 122.5; 124.5; 134.2; 137.1; 144.2; 149.4; 158.5; 161.5; 180.1. HRMS (ESI): *m/z* calculated for C_15_H_19_N_8_S: 343.1453, found: 343.1464 [M+H]^+^.

*N’-[(3-aminopyridin-2-yl)methylidene]-4-(pyrazin-2-yl)piperazine-1-carbothiohydrazide* (**L**^**12**^)

Yellow crystals; yield 70%; mp: 217–218; ^1^H-NMR (500 MHz, *d*_*6*_-DMSO, ppm): δ 3.72 (m, 4H, CH_2_), 4.09 (m, 4H, CH_2_), 7.05–7.12 (m, 2H, CH), 7.14–7.22 (s, 2H, NH_2_), 7.84 (dd, 1H; *J*_*1*_ = 4.2 Hz, *J*_*2*_ = 1.6 Hz), 7.88 (d, 1H; *J* = 2.6 Hz), 8.12 (dd, 1H; *J*_*1*_ = 2.7 Hz, *J*_*2*_ = 1.5 Hz), 8.35 (d, 1H, *J* = 1.6 Hz), 8.54 (s, 1H, CH), 11.44 (s, 1H, NH). ^13^C-NMR (126 MHz, *d*_*6*_-DMSO, ppm): δ 43.8; 48.0; 122.5; 124.5; 131.7; 133.1; 134.2; 137.2; 141.9; 144.2; 149.6; 154.7; 180.0. HRMS (ESI): *m/z* calculated for C_15_H_19_N_8_S: 343.1453, found: 343.1454 [M+H]^+^.

### Materials and physico-chemical measurements

Starting Materials and Solvents. The solution studies were carried out in bidistilled water. All the chemicals were commercial products of reagent grade and were used without further purification.

The Iron(III) stock solution was prepared from FeCl_3_·6H_2_O (Aldrich) in 1.01 × 10^−2^ M HCl (Chempur 38%) immediately before use. The Copper(II) solution was prepared from CuCl_2_ ·2H_2_O (Aldrich). All metal stock solutions were standardized using ICP-AES. The HCl solution was titrated using standardized NaOH (0.1 M Fluka standard solution).

#### pH-dependent UV-visible titrations

To determine the acid-base properties of the studied ligands, UV-visible spectrophotometric experiments as a function of p[H] were carried out in the pH range 2–11.

The isosbestic curves were used to determine the ability of a binding event of the ligand with Fe(III) and Cu(II). A series of six samples were prepared for each ligand. The ligand was dissolved in 0.1 M KCl in MeOH/H_2_O (80/20 w/w) ionic strength and mixed with a freshly prepared FeCl_3_·6H_2_O or CuCl_2_·2H_2_O solution at various ratio concentrations of the metal ions. The complex that was formed for each reaction mixture was allowed to stand for 30 min before the analysis. Absorption spectra were recorded in a Hellma quartz optical cell (1 cm) on a Jasco V630 spectrophotometer.

All the titrations were carried out on 3.2 mL samples. The metal-ligand system titrations were performed on solutions of ligand concentrations of 5x10^-5^ M and metal-to-ligand molar ratios of 1:1, 1:2, 1:3, 1:4 and 1:5.

UV-Visible titrations were also carried out. The absorption spectra (200–800 nm) were recorded using a Jasco V630 spectrophotometer. The initial pH of 3.2 mL ligand samples was adjusted to be acidic or basic and the titration of the solution was then carried out by adding known volumes of NaOH or HCl, respectively. The spectrophotometric data were fitted using the HypSpec [83] program. Distribution diagrams of the species were calculated using Hyperquad Simulation and Speciation (HySS) software [84].

### Antitumor activity

#### Cell lines

The human colon carcinoma cell line HCT116 wild type (p53^+/+^), human breast carcinoma cell line MCF-7 and glioblastoma cell line Hs683 were obtained from ATCC. The glioma cell line U-251 was purchased from Sigma Aldrich and the normal human fibroblast cell lines NHDF from PromoCell. The human colon cancer cell line HCT116 with a p53 deletion (p53^-/-^) was kindly provided by Prof. M. Rusin from the Maria Sklodowska-Curie Memorial Cancer Centre and Institute of Oncology in Gliwice, Poland. Cells were grown as monolayer cultures in Dulbecco’s modified Eagle’s medium with an antibiotic gentamicin (200 μL/100 mL medium) in 75 cm^2^ flasks (Nunc). DMEM for HCT116, MCF-7, U-251, Hs683 were supplemented with 12% heat-inactivated fetal bovine serum (Sigma) and for NHDF with 15% non-inactivated fetal bovine serum (Sigma). All the cell lines were cultured under standard conditions at 37°C in a humidified atmosphere at 5% CO_2_.

#### Cytotoxicity studies

The cells were seeded in 96-well plates (Nunc) at a density of 5,000 cells/well (HCT116, MCF-7, U-251, Hs683) and 4,000 cells/well (NHDF) and incubated at 37°C for 24 h. The assay was performed following a 72 h incubation with varying concentrations of the compounds that were tested. Then, 20 μL of The CellTiter 96®AQueous One Solution-MTS (Promega) solution was added to each well (with 100 μL DMEM without phenol red) and incubated for 1 h at 37°C. The optical densities of the samples were analyzed at 490 nm using a multi-plate reader (Synergy 4, Bio Tek). Results were expressed as a percentage of the control and calculated as the inhibitory concentration (IC_50_) values using GraphPad Prism 5. The IC_50_ parameter was defined as the compound concentration that was necessary to reduce the proliferation of cells to 50% of the untreated control. Each individual compound was tested in triplicate in a single experiment with each experiment being repeated four to five times.

#### Immunoblotting

The HCT116 (p53^+/+^), U-251 and MCF-7 cells were seeded in 3 cm Petri dishes (Nunc) at a density of 0.5·10^6^ cells/well and incubated overnight. The next day, a solution of L^9^ at a four-fold IC_50_ concentration for each cell line was added and incubated for 24 h. Cells were harvested by trypsinization, washed with cold PBS and cell pellets were obtained by centrifugation at 2,000 rpm. Total cell lysates were obtained by dissolving the cell pellets in 150 μL of a RIPA buffer (Thermo Scientific) containing a Halt Protease Inhibitor Cocktail (Thermo Scientific) or Halt Phosphatase Inhibitor Cocktail (Thermo Scientific) along with 0.5 M EDTA and lysed for 20 min on ice on a rocking plate. The lysates were then sonicated, centrifuged at 10,000 rpm for 10 min at 4°C and the supernatants were collected for further analysis. The protein concentration was determined using a Micro BCA™ Protein Assay Kit (Thermo Scientific) according to the manufacturer’s instructions. Equal amounts of proteins (15 μg) were electrophoresed on SDS-Page gels and transferred onto a nitrocellulose membranes. The membranes were blocked in 5% non-fat milk prepared in PBS containing 0.1% Tween-20 (TPBS) for 1 h. After blocking, the membranes were incubated with specific primary antibodies: PARP, p53, p21^Waf1/Cip1^, cdc2, cyclin E, cytochrome c, caspase-3,8,9 and GAPPH overnight at 4°C, then washed and incubated with horseradish peroxidase (HRP)-conjugated secondary antibodies for 1 h at room temperature. All the antibodies were purchased from Cell Signaling and were diluted 1:1000 in 5% milk in TPBS. Finally, the membranes were washed and incubated with a SuperSignal™ West Pico Chemiluminescent Substrate (Thermo Scientific). The chemiluminescence signals were captured using a ChemiDoc™ XRS+ System (BioRad). The experiments were performed at least three times.

#### Cell cycle assay

The HCT116 (p53^+/+^), U-251 and MCF-7 cells were seeded in 3 cm Petri dishes (Nunc) at a density of 0.25·10^6^ cells/well and incubated at 37°C for 24 h. Then, the medium was removed and a freshly prepared solution of the tested compound–L^9^ at a 0.5 μM concentration was added. After 48 h of treatment, assays were performed using a Muse Cell-Cycle Kit (Millipore) according to the manufacturer's instructions. Briefly, the cells were collected, washed with cold PBS and were centrifuged at 300 g. Then, the cells were fixed in ice cold 70% ethanol and stored at -20°C overnight. Afterwards, the cells were centrifuged and resuspended in 200 μL of Muse™ Cell Cycle Reagent and incubated for 30 min at room temperature in the dark. After staining, the cells were processed for cell cycle analysis using a Muse Cell Analyzer (Millipore). The experiments were performed at least three times.

#### Annexin V binding assay

The HCT116 (p53^+/+^), U-251 and MCF-7 cells were seeded in 3 cm Petri dishes (Nunc) at a density of 0.25·10^6^ cells/dish and incubated at 37°C for 24 h. After treatment with 0.5 μM of L^9^ for 48 h, the assays were performed using an Annexin V & Dead Cell Kit (Millipore) according to the manufacturer's instructions. Briefly, detached and adherent cells were collected and centrifuged at 500 g for 5 min. Next, the resuspended cells were incubated with 100 μL of Muse™ Annexin V & Dead Cell Reagent for 20 min at room temperature in the dark. After staining, the events for live, early and late apoptotic cells were counted using a Muse Cell Analyzer (Millipore). The experiments were performed at least three times.

#### Statistical analysis

All of data were expressed as the mean ± standard deviation (SD) of the results obtained from at least three independent experiments. Statistical differences was performed using one-way ANOVA with a Bonferroni post-hoc test (comparison to control). A p-value of 0.05 or less was considered to be statistically significant. GraphPad Prism v.5.0 software (GraphPad Software, USA) was used for analysis.

## Supporting information

S1 Fig The spectra and distribution forms of the ligands. (a) Absorption spectrophotometric titration vs. pH of the free L^1^, L^8^, L^11^ and L^12^ ligands; (b) electronic spectra of the protonated species; (c) concentration distribution curves for chosen ligands species. I = 0.1 M (KCl) in 80% (w/w) MeOH/H_2_O; T = 25.0°C; [L] = 5x10^-5^ M; pH 1.90–11.5.
(DOCX)

S2 Fig The effect of L9 on the expression of the proteins–full view from Western blot analysis. (A) GADPH, (B) caspase-8, (C) caspase-9, (D) GADPH, (E) p53, (F) PARP, (G) cyclin E, (H) cdc2, (I) caspase-3, (J) p21, (K) cytochrome c.
(DOCX)

S1 Table Changes in absorption spectra and isosbestic points for chosen ligands. (DOCX)

S2 Table Therapeutic indexes of the novel Triapine analogs describing their selectivity against normal cells. TI values I > 25, TI 2.5–25, TI < 2.5 were marked with red, yellow and grey color, respectively.
(DOCX)
