# Supplementary material for: Piperazinyl fragment improves anticancer activity of Triapine
Source: PLoS One. 2018 Apr 13;13(4):e0188767. doi: 10.1371/journal.pone.0188767 (PMC5898707; doi:10.1371/journal.pone.0188767)
Supplement: S1 Fig — (a) Absorption spectrophotometric titration vs. pH of the free L1, L8, L11 and L12 ligands; (b) electronic spectra of the protonated species; (c) concentration distribution curves for chosen ligands species. I = 0.1 M (KCl) in 80% (w/w) MeOH/H2O; T = 25.0°C; [L] = 5x10^-5^ M; pH 1.90–11.5. (DOCX) [file pone.0188767.s001.docx]

| (a) | (b) | (c) |
| --- | --- | --- |
|  |  |  |
|  |  |  |
|  |  |  |
|  |  |  |

**S1 Fig. The spectra and distribution forms of the ligands.** (a) Absorption spectrophotometric titration vs. pH of the free L^1^, L^8^, L^11^ and L^12^ ligands; (b) electronic spectra of the protonated species ;(c) concentration distribution curves for chosen ligands species. I = 0.1 M (KCl) in 80% (w/w) MeOH/H_2_O; T = 25.0 ^o^C; [L] = 5x10^-5^ M; pH 1.90-11.5.
