# Supplementary material for: Piperazinyl fragment improves anticancer activity of Triapine
Source: PLoS One. 2018 Apr 13;13(4):e0188767. doi: 10.1371/journal.pone.0188767 (PMC5898707; doi:10.1371/journal.pone.0188767)
Supplement: S2 Fig — (A) GADPH, (B) caspase-8, (C) caspase-9, (D) GADPH, (E) p53, (F) PARP, (G) cyclin E, (H) cdc2, (I) caspase-3, (J) p21, (K) cytochrome c. (DOCX) [file pone.0188767.s002.docx]

**
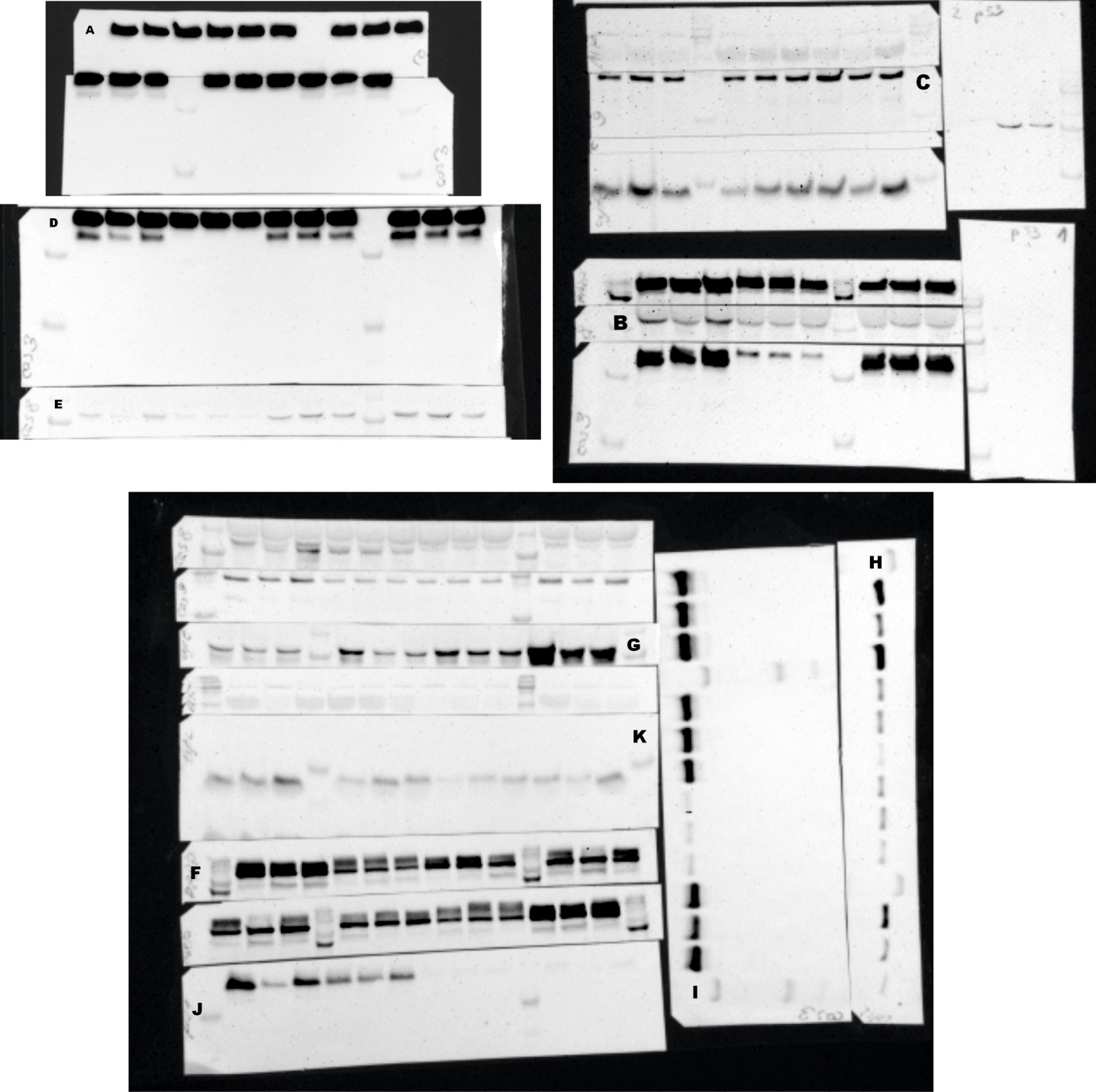
**

**S2 Fig. The effect of L^9^ on the expression of the proteins – full view from Western blot analysis.** (A) GADPH, (B) caspase-8, (C) caspase-9, (D) GADPH, (E) p53, (F) PARP, (G) cyclin E, (H) cdc2, (I) caspase-3, (J) p21, (K) cytochrome c.
