# Supplementary material for: Piperazinyl fragment improves anticancer activity of Triapine
Source: PLoS One. 2018 Apr 13;13(4):e0188767. doi: 10.1371/journal.pone.0188767 (PMC5898707; doi:10.1371/journal.pone.0188767)
Supplement: S1 Table — (DOCX) [file pone.0188767.s003.docx]

**S1 Table. Changes in absorption spectra and isosbestic points for chosen ligands.**

| **Compound** |  | **Copper** | **Iron** |
| --- | --- | --- | --- |
| **L^1^** | change in absorption after metal coordination | increase of band absorption at 247 nm and 370;  decrease the intensity of band at 450 nm | increase of band absorption at 306 nm;  decrease the intensity of band at 433 nm |
|  | isosbestic points: | 325 nm; 401 nm | 397 nm |
| **L^2^** | change in absorption after metal coordination | increase of band absorption at 370 nm;  significant decrease the intensity of band at ≈ 293 nm and 450 nm | increase of band absorption at 370 nm;  significant decrease the intensity of band at 450 nm, new visible band at 310 nm |
|  | isosbestic points: | 334 nm; 397 nm | 394 nm |
| **L^4^** | change in absorption after metal coordination | increase of band absorption at 373 nm;  significant decrease the intensity of band at ≈ 296 nm and 450 nm | increase of band absorption at 381 nm;  significant decrease the intensity of band at 436 nm, new visible band at 303 nm |
|  | isosbestic points: | 337 nm; 398 nm | 337 nm; 397 nm |
| **L^5^** | change in absorption after metal coordination | increase of band absorption at 370 nm;  significant decrease the intensity of band at 450 nm | increase of band absorption at 370 nm;  significant decrease the intensity of band at 433 nm |
|  | isosbestic points: | 336 nm; 397 nm | 393 nm |
| **L^7^** | change in absorption after metal coordination | increase of band absorption at 370 nm;  significant decrease the intensity of band at 450 nm | increase of band absorption at 269 nm and 376 nm;  significant decrease the intensity of band at 436 nm |
|  | isosbestic points: | 336 nm; 397 nm | 393 nm |
| **L^9^** | change in absorption after metal coordination | decrease the intensity of band at ≈ 298 nm and 450 nm | decrease the intensity of band at ≈ 304 nm and 430 nm |
|  | isosbestic points: | 342 nm; 395 nm | 393 nm |
| **L^12^** | change in absorption after metal coordination | increase of band absorption at 365 nm;  significant decrease the intensity of band at 450 nm | increase of band absorption at 366 nm;  significant decrease the intensity of band at 430 nm |
|  | isosbestic points: | 340 nm; 389 nm | 395 nm |
