# Supplementary material for: Piperazinyl fragment improves anticancer activity of Triapine
Source: PLoS One. 2018 Apr 13;13(4):e0188767. doi: 10.1371/journal.pone.0188767 (PMC5898707; doi:10.1371/journal.pone.0188767)
Supplement: S2 Table — TI values I > 25, TI 2.5–25, TI < 2.5 were marked with red, yellow and grey color, respectively. (DOCX) [file pone.0188767.s004.docx]

**S2 Table. Therapeutic indexes of the novel Triapine analogs describing their selectivity against normal cells.** TI values I > 25, TI 2.5-25, TI < 2.5 were marked with red, yellow and grey color, respectively.

| **Therapeutic index (TI)** | | | | | |
| --- | --- | --- | --- | --- | --- |
| Name | NHDF vs.  HCT116  p53^+/+^ | NHDF vs.  HCT116  p53^-/-^ | NHDF vs.  MCF-7 | NHDF vs.  U-251 | NHDF vs.  Hs683 |
| **L^1^** | >16 | >195 | >47 | >62 | >17 |
| **L^2^** | >48 | >164 | >69 | >28 | >10 |
| **L^3^** | >208 | >150 | >123 | >195 | >17 |
| **L^4^** | >19 | >135 | >59 | >39 | >6 |
| **L^5^** | >17 | >34 | >7 | >10 | >2 |
| **L^6^** | >180 | >93 | >53 | >66 | >13 |
| **L^7^** | >33 | >22 | >10 | >8 | >5 |
| **L^8^** | >13 | >180 | >22 | >23 | >22 |
| **L^9^** | >59 | >203 | >123 | >90 | >30 |
| **L^10^** | >147 | >157 | >97 | >182 | >128 |
| **L^11^** | >37 | >38 | >14 | >20 | >9 |
| **L^12^** | >57 | >73 | >11 | >34 | >10 |
| **L^13^/3-AP** | >22 | >19 | >11 | >17 | >14 |
